# Activity of the adrenomedullin system to personalise post-discharge diuretic treatment in acute heart failure

**DOI:** 10.1007/s00392-021-01909-9

**Published:** 2021-07-23

**Authors:** Nikola Kozhuharov, Leong Ng, Desiree Wussler, Ivo Strebel, Zaid Sabti, Oliver Hartmann, Mohamed Eltayeb, Iain Squire, Albina Nowak, Max Rieger, Jasmin Martin, Eleni Michou, Sabrina Stefanelli, Christian Puelacher, Samyut Shrestha, Maria Belkin, Tobias Zimmermann, Pedro Lopez-Ayala, Joachim Struck, Andreas Bergmann, Alexandre Mebazaa, Alice Blet, Danielle Menosi Gualandro, Tobias Breidthardt, Christian Mueller

**Affiliations:** 1 Department of Cardiology and Cardiovascular Research Institute Basel (CRIB), University Hospital Basel and University of Basel, Petersgraben 4, CH-4031 Basel, Switzerland; 2 GREAT Network, Rome, Italy; 3 Institute of Cardiovascular Medicine and Science, Liverpool Heart and Chest Hospital, Liverpool, UK; 4 Department of Cardiovascular Sciences, University of Leicester, Leicester, UK; 5 NIHR Leicester Cardiovascular Biomedical Research Unit, Glenfield Hospital, Leicester, UK; 6 Department of Internal Medicine, University Hospital Basel, University of Basel, Basel, Switzerland; 7 Sphingotec GmbH, Hennigsdorf, Germany; 8 Department of Endocrinology and Clinical Nutrition, University Hospital Zürich, University of Zürich, Zürich, Switzerland; 9 University Paris Diderot, APHP Hôpitaux Universitaires Saint Louis Lariboisière, Inserm 942, Paris, France

**Keywords:** Dyspnoea, Acute heart failure, Adrenomedullin, Diuretics

## Abstract

**Background:**

Quantifying the activity of the adrenomedullin system might help to monitor and guide treatment in acute heart failure (AHF) patients. The aims were to (1) identify AHF patients with marked benefit or harm from specific treatments at hospital discharge and (2) predict mortality by quantifying the adrenomedullin system activity.

**Methods:**

This was a prospective multicentre study. AHF diagnosis and phenotype were centrally adjudicated by two independent cardiologists among patients presenting to the emergency department with acute dyspnoea. Adrenomedullin system activity was quantified using the biologically active component, bioactive adrenomedullin (bio-ADM), and a prohormone fragment, midregional proadrenomedullin (MR-proADM). Bio-ADM and MR-proADM concentrations were measured in a blinded fashion at presentation and at discharge. Interaction with specific treatments at discharge and the utility of these biomarkers on predicting outcomes during 365-day follow-up were assessed.

**Results:**

Among 1886 patients with adjudicated AHF, 514 patients (27.3%) died during 365-day follow-up. After adjusting for age, creatinine, and treatment at discharge, patients with bio-ADM plasma concentrations above the median (> 44.6 pg/mL) derived disproportional benefit if treated with diuretics (interaction *p* values < 0.001). These findings were confirmed when quantifying adrenomedullin system activity using MR-proADM (*n* = 764) (interaction *p* values < 0.001). Patients with bio-ADM plasma concentrations above the median were at increased risk of death (hazard ratio 1.87, 95% CI 1.57–2.24; *p* < 0.001). For predicting 365-day all-cause mortality, both biomarkers performed well, with MR-proADM presenting an even higher predictive accuracy compared to bio-ADM (*p* < 0.001).

**Conclusions:**

Quantifying the adrenomedullin’s system activity may help to personalise post-discharge diuretic treatment and enable accurate risk-prediction in AHF.

**Supplementary Information:**

The online version contains supplementary material available at 10.1007/s00392-021-01909-9.

## Introduction

Acute heart failure (AHF) is the most common cause of hospitalisation in patients 50 years or older, and it is still associated with unacceptably high mortality and morbidity [1, 2], with up to 30% of patients dying within 1 year after hospital discharge [1]. The poor outcomes of patients with AHF are at least in part due to incomplete understanding of the pathophysiology of AHF, as well as uncertainty in defining the intensity of both in-hospital and immediate post-discharge management resulting from a lack of high-quality evidence for treatments in these clinical settings [1, 3].

The use of cardiovascular biomarkers may contribute to addressing some of these unmet clinical needs. For instance, the clinical introduction of natriuretic peptides quantifying haemodynamic cardiac stress has substantially improved early and accurate diagnosis of AHF [4–7]. Novel cardiovascular biomarkers may help to unravel distinct pathophysiological AHF phenotypes with marked benefits (or harm) from specific treatments and allow personalised treatment and management. Initial pilot studies suggested that the adrenomedullin system may allow quantifying systemic microvascular dysfunction and the associated mortality risk [8–13]. Specifically, adrenomedullin secretion is increased upon volume overload and is expected to function as a compensatory mechanism maintaining barrier function, thus hindering tissue congestion [14]. In this context, a clinical phase-2 trial with a monoclonal antibody against the N-terminal end of adrenomedullin (adrecizumab), which increases the plasma concentrations of bioactive adrenomedullin, has recently been initiated in AHF patients (NCT04252937) [15]. Furthermore, since residual congestion in AHF is associated with adverse outcomes, and evidence from large clinical trials guiding the use of diuretics is generally lacking, the ability of adrenomedullin to reflect tissue congestion may be particularly useful for this purpose [16].

In an international multicentre study, we aimed to use two complimentary biochemical windows to quantify the activity of the adrenomedullin system to: (1) identify AHF patients with marked benefit or harm from specific treatments at hospital discharge, and (2) predict mortality. The first biomarker used was midregional proadrenomedullin (MR-proADM), a stable precursor that does not distinguish between biologically active amidated adrenomedullin and the non-functional adrenomedullin variant containing a glycine-extended C-terminal residue [15]. The second biomarker was the biologically active form of adrenomedullin, bioactive adrenomedullin (bio-ADM) [17].

## Methods

### Study design and adjudication of final diagnosis

The study sample included three prospective diagnostic cohorts of unselected AHF patients admitted after presenting with acute dyspnoea to the emergency department (ED) of the respective participating university hospitals in three countries using comparable methodology (United Kingdom, France, and Switzerland NCT01831115). Assessment and therapies for AHF, including diuretics, were provided according to guidelines and the discretion of attending physicians [6]. AHF was centrally adjudicated by two independent cardiologists in accordance with European Society of Cardiology guidelines [6]. Inclusion was independent of renal function, although patients with terminal renal failure on renal replacement therapy were excluded. Patients were followed for at least 1 year. This study was performed according to the principles of the Declaration of Helsinki and ethics approval was granted from the respective research ethics committees. All patients provided written informed consent. The authors designed the study, gathered, and analysed the data according to The TRIPOD Statement for studies reporting multivariable prediction models for individual prognosis (Supplemental Table 1), vouch for the data and analysis, wrote the paper, and decided to publish.

### Plasma sampling

After obtaining signed informed consent, venous blood was drawn from recumbent patients and collected in tubes containing ethylenediaminetetraacetic acid as anticoagulant. The interval for sample obtainment at admission was up to 4 h (Paris), 2 h (Basel), and 12 h (Leicester) after ED presentation. Bio-ADM and MR-proADM blood samples at hospital (acute ward) discharge were available for patients recruited in Basel and Leicester. Furthermore, due to funding as well as logistics reasons, MR-proADM measurements were available only in a subgroup of unselected patients. To maximise the generalisability of the findings and considering substantial variation in overall length of hospital stay and the availability of rehabilitation units among different countries, “hospital discharge” samples were obtained at discharge from the acute ward, prior to possible transfer to a rehabilitation unit. Plasma was stored at − 80 °C until blinded analysis in a central laboratory.

### Imaging, renal function, haemoconcentration, and biomarker assays

Transthoracic echocardiography was performed using standard techniques and the left ventricular ejection fraction (LVEF) was calculated using the biplane method of discs formula. According to the LVEF, patients were stratified as heart failure with preserved ejection fraction (HFpEF, LVEF ≥ 50%), mid-range ejection fraction (HFmrEF, LVEF 40–49), and reduced ejection fraction (HFrEF, LVEF < 40%). Imaging data were available for patients recruited in Leicester and Basel. Haemoconcentration was defined as an increase in at least three of the four haemoconcentration-defining parameters (haemoglobin, haematocrit, albumin, and total protein) above admission values occurring simultaneously at discharge, as described previously [18]. These parameters as well as clinical data on volume overload such as estimated jugular venous pressure, periphery oedema, or third heart sound were only available in patients recruited in Basel. To determine plasma bio-ADM concentrations, a new double-monoclonal antibody sandwich immunoassay was used (sphingotec GmbH, Hennigsdorf, Germany). This immunoassay selectively detects the C-terminally amidated form of adrenomedullin. In healthy subjects, the median value was previously determined to be 24.7 pg/mL and the 99th percentile 43 pg/mL. The lower detection limit is 3 pg/mL, and intra- and interassay coefficients were 5–10%, and 4–8%, respectively, in the above normal measuring range. The analytical assay sensitivity is 2 ng/L [12, 19]. MR-proADM was measured using an automated sandwich chemiluminescence immunoassay on the KRYPTOR system (B·R·A·H·M·S AG, Hennigsdorf/Berlin, Germany), with a quantification limit of 0.23 nmol/L, a within-run imprecision (coefficient of variation) of 1.9%, and a between-run imprecision (coefficient of variation) of 9.8% [9, 20].

### Outcome measures

The two co-primary objectives were: (1) identification of AHF phenotypes with disproportional benefit or harm from medical treatment at discharge in terms of all-cause mortality during the 365-day follow-up. This was assessed by exploring interactions between bio-ADM plasma concentrations and treatment with diuretics, angiotensin-converting-enzyme (ACE) inhibitors or angiotensin receptor blockers (ARBs), beta blockers, and aldosterone-antagonists at discharge. (2) Assessment of the prognostic accuracy of bio-ADM for predicting all-cause mortality during the 365-day follow-up. The secondary objectives were defined as (1) and (2) with the combined outcome measure of all-cause mortality and AHF readmissions during follow-up. An additional aim was to directly compare bio-ADM with MR-proADM in these indications. Furthermore, the incremental value of both biomarkers was assessed when added to the OPTIMIZE-HF clinical risk-score, established for predicting up to 90-day all-cause mortality after hospitalisation for AHF [21]. Similarly, the independent predictive value of bio-ADM and MR-proADM was compared to the impact of haemoconcentration during hospitalisation [18].

Endpoints were ascertained blinded to biomarker data from hospital records and electronic databases. Patients who survived until discharge were followed for at least 365 days after the initial hospitalisation. Data on AHF readmissions were not available for patients from Paris (*n* = 225, 11% of the overall cohort).

### Statistical analysis

The Kolmogorov–Smirnov test and visual inspection of the distribution of variables was used for testing normality. Continuous variables are presented as medians with interquartile range, and categorical variables as numbers and percentages. Comparisons between groups were made using Chi-square, Mann–Whitney *U*, and Kruskal–Wallis tests, as appropriate. Spearman’s rho was used to analyse correlations. One-way ANOVA and Eta test statistics were used to assess associations of categorical variables and continuous variables. All-cause mortality and its combination with AHF hospitalisations during follow-up were plotted in Kaplan–Meier curves, and the log-rank test was used to assess differences between groups. The interaction *p* values between biomarker plasma concentrations and the predefined subgroups according to medication at discharge were calculated in multivariable models using Cox proportional hazards analysis. Further adjustment of these multivariable models was imposed for clinical considerations: the variables age and creatinine plasma concentrations at discharge were incorporated, as both may affect the prescription of heart failure drugs and mortality. Severe renal dysfunction and hypotension (systolic blood pressure below 90 mmHg) were considered clinical criteria, possibly justifying temporary withdrawal of diuretic therapy. In a subgroup of patients with available haemoconcentration data, this parameter as well as bio-ADM or MR-proADM and variables from a validated risk model to predict 365-day all-cause mortality were entered in multivariable regression models [22]. Sensitivity analysis was performed after excluding patients discharged from acute wards to palliative care. Hazard ratios (HR) are presented with 95% confidence intervals (CI). The prognostic accuracy of bio-ADM and MR-proADM plasma concentrations were quantified using the area under the time-dependent receiver-operating characteristic curves (AUC) and compared as described previously [23]. Furthermore, time-dependent receiver-operating characteristic curves were also used to assess the prognostic accuracy of the OPTIMIZE-HF risk-score alone and its combination with each biomarker. This was a post hoc analysis within prospective studies, and the sample size of the overall cohort was not determined specifically for this analysis [18]. No imputation was performed for missing values. Patients without complete clinical follow-up were censored at the time of the last known contact. All hypothesis testing was 2-sided and after Bonferroni correction for multiple testing (16 tests for interactions for the primary endpoint of all-cause mortality) a *p *value ≤ 0.003 was considered significant. Statistical analyses were performed using SPSS version 25, R version 3.5.1 (“timeROC”).

## Results

### Patient characteristics and biomarkers

A total of 1886 AHF patients enrolled between March 2006 and June 2015 were eligible for this analysis (Supplemental Fig. 1); 37.9% were women and the median age was 78 years (Table 1); 34.8% patients had HFpEF, 14.2% had HFmrEF, and 20.1% had HFrEF. Median bio-ADM concentrations at admission were 44.6 pg/mL, being similar between women and men (*p* = 0.219). Bio-ADM and MR-proADM concentrations correlated moderately (r = 0.639), and bio-ADM correlated less strongly with age, NT-proBNP, and serum creatinine versus MR-proADM concentrations (Table 2).

**Table 1:** Patient characteristics according to study site

	All patients (*n* = 1886)	Leicester (*n* = 862)	Paris (*n* = 215)	Basel (*n* = 809)
Demographics				
Age, years	78.0 (69.0–84.0)	77.0 (68.1–83.0)	76.0 (66.0–84.0)	79.0 (70.0–85.0)
Female, gender, %	37.9	37.4	38.6	38.2
BMI, kg/m^2^	27.4 (24.1–31.6)	31.4 (27.2–37.7)	*	26.5 (23.5–30.1)
Clinical parameters at ED				
SBP, mmHg	134 (117–153)	134 (116–151)	135.0 (113–158)	135 (118–154)
HR, beats/min	87 (72–105)	88 (73–105)	85 (69–105)	85 (71–103)
LVEF, %	39 (27–52)	35 (25–47)	*	44 (30–55)
LVEDD, cm	5.3 (4.6–5.9)	5.3 (4.7–5.9)	*	5.2 (4.6–5.9)
LVESD, cm	4.2 (3.3–5.1)	4.4 (3.6–5.2)	*	3.8 (3.0–4.9)
Medical history				
CKD, %	33.1	21.7	20.9	48.6
Hypertension, %	69.7	58.6	64.2	82.9
Dyslipidaemia, %	42.0	26.2	*	59.5
Stroke or TIA, %	16.2	16.9	*	15.3
Current or ex-smoker, %	55.4	48.0	*	63.7
Atrial fibrillation, %	42.7	47.3	*	37.8
PAD, %	10.6	5.3	*	16.4
COPD, %	16.1	10.2	*	22.5
Diabetes, %	32.0	34.6	29.3	30.0
Medication at presentation				
ACE inhibitors/ARB, %	61.5	57.0	*	66.3
Beta blockers, %	53.6	44.2	*	63.8
Aldosterone antagonists, %	13.1	12.5	*	13.7
Loop diuretics, %	65.4	61.1	*	70.0
Medication at discharge				
ACE inhibitors/ARB, %	69.6	63.9	63.7	78.1
Beta blockers, %	62.3	53.2	61.4	72.4
Aldosterone antagonists, %	31.0	32.9	26.0	30.3
Loop diuretics, %	85.0	81.3	78.1	91.1
Laboratory parameters at admission				
Haemoglobin, g/L	125.0 (111.0–138.0)	123.0 (109.0–137.0)	*	126.0 (113.0–139.0)
Sodium, mmol/L	139.0 (136.0–141.0)	138.0 (135.0–141.0)	137.0 (134.0–140.0)	139.0 (137.0–142.0)
Potassium, mmol/L	4.3 (3.9–4.6)	4.4 (4.0–4.7)	*	4.2 (3.8–4.5)
Creatinine, μmol/L	111.0 (86.0–146.0)	113.0 (91.0–142.0)	115.0 (84.3–151.0)	107.0 (82.0–148.0)
NT-proBNP, pg/mL	3094 (1468–6257)	2188 (984–4093)	*	4914 (2411–9787)
BNP, pg/mL	1244 (638–2376)	*	1244 (638–2376)	*
Urea, mmol/L	9.2 (6.6–13.4)	8.9 (6.5–12.9)	9.4 (6.8–14.6)	9.6 (6.6–13.4)
Bio-ADM, pg/mL	44.6 (30.2–69.2)	49.6 (33.8–81.2)	47.4 (33.8–82.8)	38.5 (26.3–57.4)
MR-proADM, nmol/L	1.67 (1.20–2.42)	1.74 (1.30–2.69)	1.18 (1.04–1.78)	1.62 (1.16–2.23)
Laboratory parameters at discharge				
Creatinine, μmol/L	111.0 (88.0–147.0)	113.0 (92.0–146.5)	*	108.0 (84.0–149.0)
NT-proBNP, pg/mL	1963 (861–4335)	1544 (644–2888)	*	2636 (1069–5844)
Bio-ADM, pg/mL	37.0 (25.8–55.6)	45.6 (31.6–68.9)	*	29.3 (20.0–41.6)
MR-proADM, nmol/L	1.42 (1.03–2.00)	1.67 (1.16–2.24)	*	1.28 (0.96–1.78)

*ACE* angiotensin-converting-enzyme, *ARBs* angiotensin receptor blocker, *BMI* body mass index, *bio-ADM* bioactive adrenomedullin, *CAD* coronary artery disease, *CCB* calcium channel blockers, *COPD* chronic obstructive pulmonary disease, *CKD* chronic kidney disease, *ED* emergency department, *HR* heart rate, *MR-proADM* midregional proadrenomedullin, *LV* left ventricle, *LVEDD* left ventricular end diastolic diameter, *NT-proBNP* N-terminal pro-B-type natriuretic peptide, *PAD* peripheral artery disease, *SBP* systolic blood pressure

*Data not available

**Table 2:** (A) Spearman’s rank correlation analysis between bio-ADM and MR-proADM and demographics, clinical characteristics, echocardiographic parameters, and biomarkers; (B) Eta test statistics to assess association between bio-ADM and MR-proADM and clinical characteristics

(A)	Bio-ADM	MR-proADM	NT-proBNP
Age, years			
Spearman’s rank (rs)	− 0.058	0.264	0.217
*p* value	0.012	< 0.001	< 0.001
*n*	1885	764	1576
BMI, kg/m^2^			
Spearman’s rank (rs)	0.295	− 0.021	− 0.385
*p* value	< 0.001	.596	< 0.001
*n*	1005	621	923
Pulse oximetry, %			
Spearman’s rank (rs)	− 0.075	− 0.044	− 0.081
*p* value	0.036	0.373	.031
*n*	786	416	710
HR, bpm			
Spearman’s rank (rs)	0.001	− 0.068	− 0.012
*p* value	0.981	0.063	0.643
*n*	1814	755	1522
SBP, mmHg			
Spearman’s rank (rs)	− 0.184	− 0.248	− 0.137
*p* value	< 0.001	< 0.001	< 0.001
*n*	1815	750	1519
DBP, mmHg			
Spearman’s rank (rs)	− 0.109	− 0.188	− 0.008
*p* value	< 0.001	< 0.001	0.769
*n*	1811	750	1516
LV ejection fraction, %			
Spearman’s rank (rs)	− 0.123	− 0.127	− 0.222
*p* value	< 0.001	0.003	< 0.001
*n*	1302	542	1234
LVEDD, mm*			
Spearman’s rank (rs)	0.057	0.046	0.144
*p* value	0.049	0.304	< 0.001
*n*	1209	505	1153
Haemoglobin, g/L			
Spearman’s rank (rs)	− 0.131	− 0.272	− 0.111
*p* value	< 0.001	< 0.001	< 0.001
*n*	1567	728	1496
Sodium, mmol/L			
Spearman’s rank (rs)	− 0.133	− 0.096	0.015
*p* value	< 0.001	.008	0.542
*n*	1855	747	1557
Creatinine, μmol/L			
Spearman’s rank (rs)	0.365	0.639	0.327
*p* value	< 0.001	< 0.001	< 0.001
*n*	1855	749	1561
NT-proBNP, pg/mL			
Spearman’s rank (rs)	0.089	0.477	–
*p* value	< 0.001	< 0.001	
*n*	1576	699	
MR-proADM, nmol/L			
Spearman’s rank (rs)	0.639	–	
*p* value	< 0.001		
*n*	764		
(B)			
Elevated JVP*			
Eta squared (*η*^2^)	0.011	0.047	< 0.001
*p* value	0.004	< 0.001	0.887
*n*	728	396	651
Third heart sound (S3)*			
Eta squared (*η*^2^)	0.003	0.005	< 0.001
*p* value	0.151	0.151	0.822
*n*	756	406	681
Peripheral oedema*			
Eta squared (*η*^2^)	0.053	0.055	0.001
*p* value	< 0.001	< 0.001	0.473
*n*	792	421	711

Correlation was calculated with measurements obtained on admission

*bio-ADM* bioactive adrenomedullin, *BMI* body mass index, *DBP* diastolic blood pressure, *LV* left ventricle, *LVEDD* left ventricular end diastolic diameter, *MR-proADM* midregional proadrenomedullin, *SBP* systolic blood pressure, *JVP* jugular venous pressure, *MR-proADM* midregional proadrenomedullin

*Data available only for patients from Basel

### Survival analysis

During the 365-day follow-up, there were 514 deaths (27.3%). Mortality was comparable among sites (Supplemental Figs. 2 and 3). Patients who died during follow-up were older, had a lower body mass index, more often had a history of renal impairment, and lower initial systolic blood pressure and LVEF (Table 3). Notably, their bio-ADM and MR-proADM plasma concentrations at admission and at hospital discharge were substantially higher compared to survivors.

**Table 3:** Patient’s characteristics according to survival status at 365 days

	Dead at 365 days (*n* = 514)	Alive at 365 days (*n* = 1372)	*p* value
Demographics			
Age, years	81.0 (75.0–87.0)	76.5 (67.0–83.0)	< 0.001
Female, gender, %	37.0	38.2	0.684
BMI, kg/m^2^	24.9 (21.8–28.4)	28.1 (24.6–32.8)	< 0.001
Clinical parameters at ED			
SBP, mmHg	126 (110–145)	137 (120–155)	< 0.001
HR, beats/min	85 (72–101)	87 (72–105)	0.159
LV ejection fraction, %	35 (24–49)	40 (29–54)	< 0.001
LVEDD, cm	5.3 (4.6–6.1)	5.2 (4.6–5.9)	0.313
LVESD, cm	4.2 (3.4–5.2)	4.2 (3.3–5.0)	0.326
Medical history			
CKD, %	44.3	28.9	< 0.001
Hypertension, %	67.7	70.4	0.280
Dyslipidaemia, %	40.7	42.4	0.560
Stroke or TIA, %	21.3	14.3	< 0.001
Current or ex-smoker, %	57.5	54.6	0.330
PAD, %	13.0	9.7	0.065
Atrial fibrillation, %	41.4	43.1	0.568
COPD, %	17.9	15.5	0.260
Diabetes, %	31.7	32.1	0.911
Medication at presentation			
ACE inhibitors or ARB, %	59.2	62.3	0.284
Beta blockers, %	52.1	54.1	0.511
Aldosterone antagonists, %	16.9	11.7	0.006
Loop diuretics, %	77.3	61.0	< 0.001
Medication at discharge			
ACE inhibitors or ARB, %	51.6	76.3	< 0.001
Beta blockers, %	45.8	68.3	< 0.001
Aldosterone antagonists, %	25.7	33.0	0.003
Loop diuretics, %	75.8	88.3	< 0.001
Laboratory parameters at admission			
Haemoglobin, g/L	120 (108–132)	127 (113–139)	< 0.001
Sodium, mmol/L	138 (134–141)	139 (136–141)	< 0.001
Potassium, mmol/L	4.4 (3.9–4.8)	4.2 (3.9–4.6)	0.006
Creatinine, μmol/L	132 (100.0–179.0)	105.0 (83.0–134.0)	< 0.001
Urea, mmol/L	12.3 (8.7–17.7)	8.4 (6.2–11.7)	< 0.001
NT-proBNP, pg/mL	4723.0 (2360–9029)	2629.0 (1259.0–5346)	< 0.001
BNP, pg/mL	1897 (810–3036)	1120 (605–2128)	0.009
Bio-ADM, pg/mL	57.2 (35.3–94.3)	41.5 (28.8–62.0)	< 0.001
MR-proADM, nmol/L	2.32 (1.68–3.07)	1.50 (1.14–2.14)	< 0.001
Laboratory parameters at discharge			
Creatinine, μmol/L	132.0 (100.0–191.0)	107.0 (85.0–135.0)	< 0.001
NT-proBNP, pg/mL	3327 (1678–8348)	1659 (686–3417)	< 0.001
Bio-ADM, pg/mL	46.7 (29.0–72.5)	34.74 (25.2–51.7)	< 0.001
MR-proADM, nmol/L	1.79 (1.45–2.77)	1.29 (0.99–1.83)	< 0.001

*ACE* angiotensin-converting-enzyme, *ARBs* angiotensin receptor blocker, *BMI* body mass index, *bio-ADM* bioactive adrenomedullin, *CAD* coronary artery disease, *CCB* calcium channel blockers, *COPD* chronic obstructive pulmonary disease, *CKD* chronic kidney disease, *ED* emergency department, *HR* heart rate, *MR-proADM* midregional proadrenomedullin, *LVEF* left ventricular ejection fraction, *LVEDD* left ventricular end diastolic diameter, *NT-proBNP* N-terminal pro-B-type natriuretic peptide, *PAD* peripheral artery disease, *SBP* systolic blood pressure

### Interaction with heart failure treatment at discharge

**Fig. 1:**
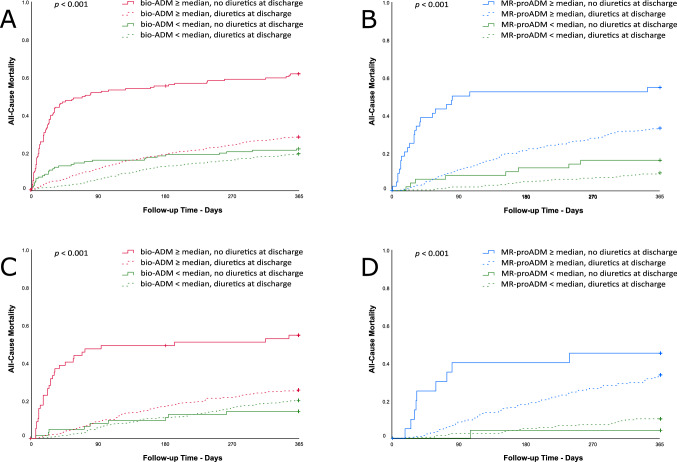
Mortality stratified according to bio-ADM and MR-pro-ADM concentration and the use of diuretics at discharge: **A** bio-ADM at presentation (*n* = 1844); **B** MR-proADM at presentation (*n* = 738); **C** bio-ADM at discharge (*n* = 997); **D** MR-proADM at discharge (*n* = 438). *ACE* angiotensin-converting-enzyme, *ARBs* angiotensin receptor blocker, *bio-ADM* bioactive adrenomedullin, *MR-proADM* midregional proadrenomedullin

There were statistically significant interactions regarding mortality depending on MR-proADM or bio-ADM plasma concentrations at admission or discharge and prescription of diuretics at discharge (Supplemental Table 2). These interactions remained significant after adjusting for age, NT-proBNP and creatinine plasma concentrations at discharge (Table 4). Overall, 276 (15%) patients were discharged without prescription of diuretics (Supplemental Table 3). Among them, history of chronic kidney disease was less common as compared to those with diuretics prescription at discharge (26.5% vs 33.9%, *p* = 0.017). Similarly, treatment with diuretics prior to admission was less common (53.1% vs 67.2%, *p* < 0.001). Patients not receiving diuretics at discharge and with bio-ADM levels above the median presented much higher all-cause mortality (Fig. 1). Findings regarding 365-day all-cause mortality and AHF rehospitalisations were comparable (Supplemental Tables 4 and 5; Supplemental Figs. 4 and 5). Furthermore, subgroup analyses according to LVEF groups suggested more pronounced interactions in patients with HFrEF (Supplemental Tables 6 and 7).

**Table 4:** Interaction *p* values in multivariable models using Cox proportional hazard analysis for predicting 365-day all-cause mortality including age, bio-ADM or MR-proADM, NT-proBNP at discharge, creatinine at discharge, and medication at discharge

	Diuretics	ACE inhibitors or ARB	Beta blockers	Aldosterone antagonists
lg bio-ADM at admission, pg/mL	**< 0.001**	0.104	0.541	0.512
lg bio-ADM at discharge, pg/mL	**< 0.001**	0.008	0.609	0.354
lg MR-proADM at admission, nmol/L	0.112	0.404	0.724	0.141
lg MR-proADM at discharge, nmol/L	**0.002**	0.225	0.969	0.381

*ACE* angiotensin-converting-enzyme, *ARBs* angiotensin receptor blocker, *bio-ADM* bioactive adrenomedullin, *MR-proADM* midregional proadrenomedullin, *NT-proBNP* N-terminal pro-B-type natriuretic peptide

The bold p values are the ones considered significant, as reported in the Methods section: All hypothesis testing was 2-sided and after Bonferroni correction for multiple testing (16 tests for interactions for the primary endpoint of all-cause mortality) a p value ≤ 0.003 was considered significant. Please include the footnote: The bold p values (≤ 0.003) are considered significant.

Notably, no statistically significant interactions were present between NT-proBNP plasma concentrations and medical therapies at discharge (Supplemental Tables 8 and 9). In-depth review of clinical characteristics of patients with bio-ADM concentrations above the median and without diuretics at discharge, who died during follow-up, showed that at acute ward discharge many did not have an established medical cause such as severe renal dysfunction or hypotension, possibly justifying withdrawal of diuretics (Supplemental Table 10).

### Prognostic accuracy of bio-ADM and MR-proADM

**Fig. 2:**
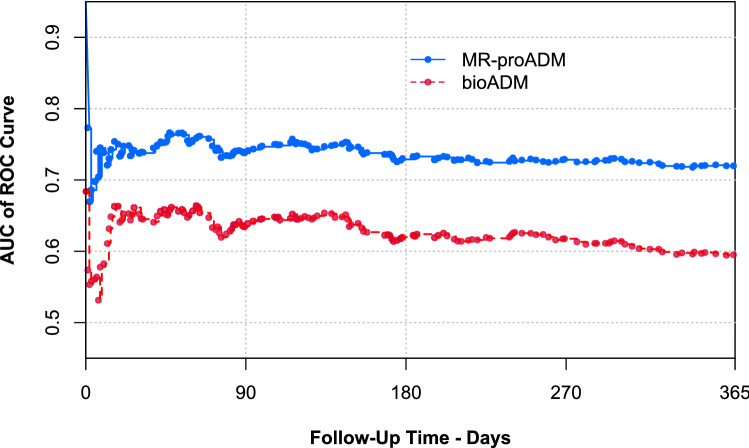
Time-dependent ROC curves describing the prognostic performance of bio-ADM and MR-proADM to predict death during 365-day follow-up (*n* = 764). AUC of ROC curve: area under the time-dependent receiver-operating characteristic curve. *bio-ADM* bioactive adrenomedullin, *MR-proADM* midregional proadrenomedullin

In 764 patients (41%), both MR-proADM and bio-ADM were measured. The prognostic accuracy for predicting all-cause mortality and the combination of all-cause mortality or AHF rehospitalisations at 365 days, quantified by the AUC, was significantly higher for admission MR-proADM compared to bio-ADM (Fig. 2 and Supplemental Fig. 6; for both outcome measures: *p* < 0.001 at 90, 180, and 365 days). Patients with bio-ADM concentrations above the median at admission were at increased risk of 365-day all-cause mortality (HR 1.87, 95% CI 1.57–2.24; *p* < 0.001). In the subgroup analysis of patients with MR-proADM measurements at admission, plasma concentrations of this biomarker above the median (> 1.67 nmol/L) were associated with a higher 365-day all-cause mortality (HR 3.88, 95% CI: 2.77–5.44; *p* < 0.001; Supplemental Fig. 7). The OPTIMIZE-HF risk-score was calculated in 1706 patients (90%), showing significant improvement in prognostic accuracy when combined with bio-ADM or MR-proADM along 90 days of follow-up (Supplemental Figs. 8 and 9). Variables from a validated risk model to predict all-cause mortality at 365 days as well as haemoconcentration achieved during hospitalisation and bio-ADM or MR-proADM were entered in multivariable regression models (Supplemental Tables 10A and 10B). Notably, both bio-ADM and MR-proADM remained independent predictors of 365-day all-cause mortality.

### Sensitivity analysis

Sensitivity analysis excluding the subgroup of patients discharged from the acute ward to palliative care (*n* = 10) revealed similar findings as in the overall cohort (Supplemental Tables 11–17 and Supplemental Figs. 10–16).

## Discussion

In this large international multicentre study, we used plasma concentrations of bio-ADM and MR-proADM to identify AHF phenotypes with disproportional benefit or harm from specific heart failure treatments at hospital discharge and to predict mortality. We report five major findings.

First, patients with bio-ADM concentrations above the median had much higher mortality if not treated with diuretics at discharge. Second, medical reasons including severe renal dysfunction and/or hypotension which could possibly justify temporal withdrawal of diuretics were absent in most of these patients. This confirms the possible clinical utility of this marker to avoid medical errors such as the withdrawal of diuretics in patients who very likely derive benefits from these drugs. Third, increased bio-ADM concentrations were associated with increased risk of death at 365 days. Notably, bio-ADM remained an independent prognostic factor for all-cause mortality after adjusting for haemoconcentration, comorbidities, vital signs, and further biomarkers from a validated multivariable prediction model [22]. Fourth, comparison of the two biochemical methods assessing adrenomedullin system activity showed that MR-proADM was superior to bio-ADM in the prediction of death and/or AHF re-hospitalisation within 365 days. Fifth, both bio-ADM and MR-proADM improved the prognostic accuracy of the OPTIMIZE-HF clinical risk-score for predicting all-cause mortality within 90 days after AHF hospitalisation.

These findings extend and corroborate earlier studies investigating the importance of adrenomedullin in patients with acute dyspnoea, as well as efforts to personalise treatment in AHF patients [9, 12, 13, 17, 24–27]. The observation that patients with high bio-ADM concentrations had much higher mortality rates if not treated with diuretics at discharge seems to have immediate clinical consequences. In-depth review of the clinical characteristics of these patients indicates inappropriate clinical reaction to temporary worsening of renal function during AHF treatment, as well as a lack of reassessment of eligibility for diuretic therapy after appropriate temporal withdrawal when arterial hypovolemia is considered the most likely cause of the absence of diuretic therapy at discharge [18, 28, 29]. Whenever medical reasons possibly justifying temporal withdrawal of diuretics, such as severe renal dysfunction or hypotension, are no longer present at discharge and in the vulnerable transition period immediately after discharge, reinstitution of diuretics seems to have major importance particularly in patients identified to be at very high risk of death by elevated bio-ADM concentrations [17, 30]. The value of bio-ADM in this setting is reinforced by the challenge to document euvolemia in hospitalised AHF patients [12]. As therapy for AHF at discharge often remains unchanged for several weeks and even months after discharge [1, 31], over-restrictive use of diuretics at this critical time point seems to have detrimental consequences for patients.

The statistically significant interaction of bio-ADM and MR-proADM with benefits from diuretics at discharge is well in line with recent evidence from a large international study characterising bio-ADM concentrations as a quantitative marker of residual congestion in heart failure [12, 30]. Interestingly, the link between bio-ADM and residual tissue congestion and/or benefit from diuretics after discharge seems to be stronger compared to that for NT-proBNP, which mainly reflects increased left ventricular filling pressures and intravascular volume overload [12]. This might be related to the important role of the adrenomedullin system in maintaining endothelial barrier function through regulation of the cortical actin formation in endothelial cells [15, 32, 33]. In vitro analysis showed that dysfunction of the adrenomedullin system leads to enhanced vascular permeability, and subsequently, pronounced oedema [33]. Accordingly, the unique ability of adrenomedullin to reflect this specific pathophysiological pathway in AHF could explain its incremental value to the established OPTIMIZE-HF clinical risk-prediction score [21].

The reason why these two biochemical approaches providing insight into the adrenomedullin system showed different results in the prediction of death and in the identification of patients who would most benefit from diuretic therapy at discharge remains largely unknown. The biological active bio-ADM was superior in the latter aspect as not only discharge, but already admission concentrations allowed the identification of patients with particular benefit from long-term diuretic therapy. In contrast, MR-proADM was superior in predicting death which might be explained by the stronger correlation of MR-proADM with age and creatinine values compared to bio-ADM.

The current study has several limitations. First, our findings were based on a large number of patients prospectively enrolled and hospitalised in three European countries. Further research is warranted to validate our findings in other European and non-European populations. Second, our results cannot be extrapolated to patients with terminal renal failure undergoing long-term haemodialysis, because they were excluded. Third, although patients were enrolled prospectively at all three sites, some variables including biomarker measurements at discharge were not available for patients enrolled in Paris. Fourth, data on dose of diuretics as well as doses of guidelines directed medical treatment for chronic heart failure was not available. Fifth, we could not assess the possible interaction between bio-ADM and MR-proADM concentrations and sacubitril/valsartan, as this drug was not yet in clinical use at patients’ enrolment. Sixth, despite in-depth review of the individual patient’s characteristics including severe renal dysfunction and/or hypotension, the final reasons why AHF patients were not prescribed with diuretics at discharge cannot be established. Seventh, statistically significant interaction does not prove causality. Therefore, only a randomised controlled intervention study can prove improved outcomes with the use of diuretics post-discharge in AHF patients guided by adrenomedullin system activity.

In conclusion, quantification of adrenomedullin system activity seems to enable accurate risk-prediction in AHF patients and identify inadequate decongestion prior to discharge, thereby has the potential to facilitate personalised post-discharge diuretic treatment.

## Supplementary Information

Below is the link to the electronic supplementary material.

Supplementary file1 (DOCX 1145 KB)

## Data Availability

The data that support the findings of this study are available from the corresponding author upon reasonable request.
