# Supplementary material for: Activity of the adrenomedullin system to personalise post-discharge diuretic treatment in acute heart failure
Source: Clin Res Cardiol. 2021 Jul 23;111(6):627–37. doi: 10.1007/s00392-021-01909-9 (PMC9151518; doi:10.1007/s00392-021-01909-9)
Supplement: Supplementary file 1 — Supplementary file1 (DOCX 1145 KB) [file 392_2021_1909_MOESM1_ESM.docx]

**Activity of the Adrenomedullin System to Personalize Post-Discharge Diuretic Treatment in Acute Heart Failure**

**Supplemental materials**

# **Tables**

**Supplemental Table 1.** TRIPOD Checklist for prediction model development.

| **Section/Topic** | **Item** | **Checklist Item** | **Page** |
| --- | --- | --- | --- |
| **Title and abstract** | | | |
| Title | 1 | Identify the study as developing and/or validating a multivariable prediction model, the target population, and the outcome to be predicted. | 1 |
| Abstract | 2 | Provide a summary of objectives, study design, setting, participants, sample size, predictors, outcome, statistical analysis, results, and conclusions. | 2 |
| **Introduction** | | | |
| Background and objectives | 3a | Explain the medical context (including whether diagnostic or prognostic) and rationale for developing or validating the multivariable prediction model, including references to existing models. | 4, 5 |
|  | 3b | Specify the objectives, including whether the study describes the development or validation of the model or both. | 5 |
| **Methods** | | | |
| Source of data | 4a | Describe the study design or source of data (e.g., randomized trial, cohort, or registry data), separately for the development and validation data sets, if applicable. | 6 |
|  | 4b | Specify the key study dates, including start of accrual; end of accrual; and, if applicable, end of follow-up. |  |
| Participants | 5a | Specify key elements of the study setting (e.g., primary care, secondary care, general population) including number and location of centres. | 6 |
|  | 5b | Describe eligibility criteria for participants. | 6 |
|  | 5c | Give details of treatments received, if relevant. | 6 |
| Outcome | 6a | Clearly define the outcome that is predicted by the prediction model, including how and when assessed. | 8, 9 |
|  | 6b | Report any actions to blind assessment of the outcome to be predicted. | 8, 9 |
| Predictors | 7a | Clearly define all predictors used in developing or validating the multivariable prediction model, including how and when they were measured. | 7, 8 |
|  | 7b | Report any actions to blind assessment of predictors for the outcome and other predictors. | 6-8 |
| Sample size | 8 | Explain how the study size was arrived at. | 9 |
| Missing data | 9 | Describe how missing data were handled (e.g., complete-case analysis, single imputation, multiple imputation) with details of any imputation method. | 9 |
| Statistical analysis methods | 10a | Describe how predictors were handled in the analyses. | 9 |
|  | 10b | Specify type of model, all model-building procedures (including any predictor selection), and method for internal validation. | 8, 9 |
|  | 10d | Specify all measures used to assess model performance and, if relevant, to compare multiple models. | 9 |
| Risk groups | 11 | Provide details on how risk groups were created, if done. |  |

| **Results** | | | |
| --- | --- | --- | --- |
| Participants | 13a | Describe the flow of participants through the study, including the number of participants with and without the outcome and, if applicable, a summary of the follow-up time. A diagram may be helpful. | 11 |
|  | 13b | Describe the characteristics of the participants (basic demographics, clinical features, available predictors), including the number of participants with missing data for predictors and outcome. | 11 |
| Model development | 14a | Specify the number of participants and outcome events in each analysis. | 11, 12 |
|  | 14b | If done, report the unadjusted association between each candidate predictor and outcome. |  |
| Model specification | 15a | Present the full prediction model to allow predictions for individuals (i.e., all regression coefficients, and model intercept or baseline survival at a given time point). | 11, 12 |
|  | 15b | Explain how to use the prediction model. |  |
| Model performance | 16 | Report performance measures (with CIs) for the prediction model. | 11, 12 |
| **Discussion** | | | |
| Limitations | 18 | Discuss any limitations of the study (such as nonrepresentative sample, few events per predictor, missing data). | 16 |
| Interpretation | 19b | Give an overall interpretation of the results, considering objectives, limitations, and results from similar studies, and other relevant evidence. | 14-16 |
| Implications | 20 | Discuss the potential clinical use of the model and implications for future research. | 15, 16 |
| **Other information** | | | |
| Supplementary information | 21 | Provide information about the availability of supplementary resources, such as study protocol, Web calculator, and data sets. | Supplementary materials |
| Funding | 22 | Give the source of funding and the role of the funders for the present study. | 17 |

**Supplemental Table 2.** Interaction *p*-values in bivariable models using a cox proportional hazard analysis for predicting all-cause mortality at 365 days including one biomarker and medication at discharge.

|  | **Diuretics** | **ACE inhibitors or ARB** | **Beta blockers** | **Aldosterone antagonists** |
| --- | --- | --- | --- | --- |
| lg bio-ADM at admission, pg/mL | **< 0.001** | 0.003 | 0.097 | **0.013** |
| lg bio-ADM at discharge, pg/mL | **< 0.001** | 0.016 | 0.488 | 0.175 |
| lg MR-proADM at admission, nmol/L | 0.046 | 0.215 | 0.107 | 0.587 |
| lg MR-proADM at discharge, nmol/L | **0.001** | 0.152 | 0.601 | 0.060 |

ACE: Angiotensin-converting-enzyme. ARBs: Angiotensin receptor blocker. bio-ADM: bioactive adrenomedullin. MR-proADM: midregional proadrenomedullin.

**Supplemental Table 3.** Patient’s characteristics according to discharge prescription of diuretics.

|  | **Patients discharged without diuretics**  **(n = 276*)** | **Patients discharged with diuretics**  **(n = 1,568*)** | ***p*-value** |
| --- | --- | --- | --- |
| **Demographics** |  |  |  |
| Age, years | 76.0 (66.0-84.0) | 78.0 (69.0-84.0) | 0.097 |
| Female gender, % | 38.5 | 33.5 | 0.110 |
| BMI, kg/m^2^ | 26.6 (23.7-30.8) | 27.7 (24.1-31.8) | 0.184 |
| **Clinical parameters at ED** |  |  |  |
| SBP, mmHg | 134 (113-152) | 134 (118-153) | 0.214 |
| HR, beats/min | 81 (69-106) | 85 (71-103) | 0.707 |
| LV Ejection Fraction, % | 35 (26-450) | 40 (27-53) | 0.176 |
| LVEDD, cm | 5.4 (4.6-6.0) | 5.2 (4.6-5.9) | 0.470 |
| LVESD, cm | 4.4 (3.2-5.3) | 4.2 (3.3-5.0) | 0.225 |

|  | **Patients discharged without diuretics**  **(n = 276*)** | **Patients discharged with diuretics**  **(n = 1,568*)** | ***p*-value** |
| --- | --- | --- | --- |
| **Medical history** |  |  |  |
| CKD, % | 26.5 | 33.9 | 0.017 |
| Hypertension, % | 60.9 | 71.0 | 0.001 |
| Dyslipidaemia, % | 33.6 | 42.8 | 0.009 |
| Stroke or TIA, % | 18.8 | 15.6 | 0.228 |
| Current or ex-smoker, % | 54.4 | 55.2 | 0.837 |
| PAD, % | 6.6 | 11.1 | 0.037 |
| Atrial fibrillation, % | 41.8 | 43.1 | 0.711 |
| COPD, % | 11.8 | 16.5 | 0.072 |
| Diabetes, % | 33.0 | 31.9 | 0.716 |
| **Medication at presentation** |  |  |  |
| ACE inhibitors or ARB, % | 57.6 | 62.7 | 0.143 |
| Beta blockers, % | 46.5 | 54.4 | 0.026 |
| Aldosterone antagonists, % | 10.9 | 13.6 | 0.260 |
| Loop diuretics, % | 53.1 | 67.2 | < 0.001 |
|  | **Patients discharged without diuretics**  **(n = 276*)** | **Patients discharged with diuretics**  **(n = 1,568*)** | ***p*-value** |
| **Medication at discharge** | |  |  |
| ACE inhibitors or ARB, % | 26.1 | 79.0 | < 0.001 |
| Beta blockers, % | 27.2 | 69.5 | < 0.001 |
| Aldosterone antagonists, % | 10.9 | 35.0 | < 0.001 |
| **Laboratory parameters at admission** | |  |  |
| Haemoglobin, g/L | 127 (113-142) | 124 (110-137) | 0.021 |
| Sodium, mmol/L | 138 (135-141) | 139 (136-141) | 0.031 |
| Potassium, mmol/L | 4.3 (3.9-4.8) | 4.3 (3.9-4.6) | 0.075 |
| Creatinine, μmol/L | 112 (85.0-150.0) | 111.0 (89.0-145.0) | 0.838 |
| Urea, mmol/L | 8.9 (6.4-14.1) | 9.7 (6.9-14.3) | 0.778 |
| NT-proBNP, pg/mL | 2,449 (1,046-5,585) | 3,145 (1,493-6,108) | 0.008 |
| Bio-ADM, pg/mL | 45.6 (28.7-79.6) | 44.4 (30.3-67.9) | 0.558 |
| MR-proADM, nmol/L | 1.54 (0.94- 2.62) | 1.67 (1.21-2.32) | 0.202 |
|  |  |  |  |
|  | **Patients discharged without diuretics**  **(n = 276*)** | **Patients discharged with diuretics**  **(n = 1,568*)** | ***p*-value** |
| **Laboratory parameters at discharge** | |  |  |
| Haemoglobin, g/L | 124 (106-136) | 121 (108-134) | 0.242 |
| Sodium, mmol/L | 139 (136-141) | 139 (136-141) | 0.129 |
| Potassium, mmol/L | 4.1 (3.8-4.5) | 4.10 (3.8-4.5) | 0.402 |
| Urea, mmol/L | 9.5 (6.3-16.3) | 9.7 (6.9-14.3) | 0.967 |
| Creatinine, μmol/L | 108.0 (84.0-157.0) | 111.0 (89.0-145.0) | 0.815 |
| NT-proBNP, pg/mL | 1,655 (651-3,160) | 1,960 (882-4,280) | 0.015 |
| Bio-ADM, pg/mL | 35.3 (23.3-73.1) | 37.1 (26.1-54.6) | 0.719 |
| MR-proADM, nmol/L | 1.30 (0.81-1.86) | 1.43 (1.07-1.99) | 0.148 |

ACE: Angiotensin-converting-enzyme. ARBs: Angiotensin receptor blocker. BMI: Body mass index. bio-ADM: bioactive adrenomedullin.

CAD: Coronary artery disease. CCB: Calcium channel blockers. COPD: Chronic obstructive pulmonary disease. CKD: Chronic kidney disease. ED: Emergency department. HR: Heart rate. MR-proADM: midregional proadrenomedullin. LVEF: Left ventricular ejection fraction. LVEDD: Left ventricular end diastolic diameter. NT-proBNP: N-terminal pro-B-type natriuretic peptide. PAD: Peripheral artery disease. SBP: Systolic blood pressure.

*In 42 of the overall cohort of 1886 patients, there was no information on prescription of diuretics at discharge

**Supplemental Table 4.** Interaction *p*-values in bivariable models using a cox proportional hazard analysis for predicting all-cause mortality or AHF hospitalizations at 365 days including one biomarker, and medication at discharge.

|  | **Diuretics** | **ACE inhibitors or ARB** | **Beta blockers** | **Aldosterone antagonists** |
| --- | --- | --- | --- | --- |
| lg bio-ADM at admission, pg/mL | **< 0.001** | 0.008 | 0.254 | 0.244 |
| lg bio-ADM at discharge, pg/mL | **< 0.001** | 0.023 | 0.732 | 0.509 |
| lg MR-proADM at admission, nmol/L | 0.011 | 0.045 | 0.019 | 0.542 |
| lg MR-proADM at discharge, nmol/L | **<0.001** | 0.011 | 0.389 | 0.285 |

ACE: Angiotensin-converting-enzyme. AHF: Acute heart failure. ARBs: Angiotensin receptor blocker. bio-ADM: bioactive adrenomedullin. MR-proADM: midregional proadrenomedullin.

**Supplemental Table 5.** Interaction *p*-values in multivariable models using a cox proportional hazard analysis for predicting all-cause mortality or AHF hospitalizations at 365 days including age, bio-ADM or MR-proADM, NT-proBNP at discharge, creatinine at discharge, and medication at discharge.

|  | **Diuretics** | **ACE inhibitors or ARB** | **Beta blockers** | **Aldosterone antagonists** |
| --- | --- | --- | --- | --- |
| lg bio-ADM at admission, pg/mL | **< 0.001** | 0.186 | 0.615 | 0.687 |
| lg bio-ADM at discharge, pg/mL | **< 0.001** | 0.015 | 0.007 | 0.699 |
| lg MR-proADM at admission, nmol/L | 0.044 | 0.263 | 0.254 | 0.069 |
| lg MR-proADM at discharge, nmol/L | **0.001** | 0.036 | 0.391 | 0.580 |

ACE: Angiotensin-converting-enzyme. AHF: Acute heart failure. ARBs: Angiotensin receptor blocker. bio-ADM: bioactive adrenomedullin. MR-proADM: midregional proadrenomedullin. NT-proBNP: N-terminal pro-B-type natriuretic peptide. **Supplemental Table 6**. Interaction p-values in bivariable models using a cox proportional hazard analysis for predicting all-cause mortality at 365 days including bio-ADM or MR-proADM, and **diuretics at discharge** according to ejection fraction group.

|  | **HFrEF** | **HFmrEF** | **HFpEF** |
| --- | --- | --- | --- |
| lg bio-ADM at admission, pg/mL | **< 0.001** | 0.834 | 0.142 |
| lg bio-ADM at discharge, pg/mL | **0.003** | 0.087 | 0.031 |
| lg MR-proADM at admission, nmol/L | 0.011 | 0.248 | 0.224 |
| lg MR-proADM at discharge, nmol/L | 0.014 | 0.414 | 0.888 |

bio-ADM: bioactive adrenomedullin. HFmrEF: heart failure with mid-range ejection fraction. HFpEF: heart failure with preserved ejection fraction. HFrEF: heart failure with reduced ejection fraction. MR-proADM: midregional proadrenomedullin.

**Supplemental Table 7**. Interaction p-values in multivariable models using a cox proportional hazard analysis for predicting all-cause mortality at 365 days including age, bio-ADM or MR-proADM, NT-proBNP at discharge, creatinine at discharge, and **diuretics at discharge** according to ejection fraction group.

|  | **HFrEF** | **HFmrEF** | **HFpEF** |
| --- | --- | --- | --- |
| lg bio-ADM at admission, pg/mL | **< 0.001** | 0.806 | 0.181 |
| lg bio-ADM at discharge, pg/mL | 0.026 | 0.278 | 0.052 |
| lg MR-proADM at admission, nmol/L | 0.043 | 0.171 | 0.400 |
| lg MR-proADM at discharge, nmol/L | 0.019 | 0.331 | 0.889 |

bio-ADM: bioactive adrenomedullin. HFmrEF: heart failure with mid-range ejection fraction. HFpEF: heart failure with preserved ejection fraction. HFrEF: heart failure with reduced ejection fraction. MR-proADM: midregional proadrenomedullin. NT-proBNP: N-terminal pro-B-type natriuretic peptide.

**Supplemental Table 8.** Interaction *p*-values in multivariable models using a cox proportional hazard analysis for predicting all-cause mortality at 365 days including age, NT-proBNP, creatinine at discharge, and medication at discharge.

|  | **Diuretics** | **ACE inhibitors or ARB** | **Beta blockers** | **Aldosterone antagonists** |
| --- | --- | --- | --- | --- |
| lg NT-proBNP at admission, pg/mL | 0.428 | 0.914 | 0.244 | 0.709 |
| lg NT-proBNP at discharge, pg/mL | 0.009 | 0.307 | 0.237 | 0.575 |

ACE: Angiotensin-converting-enzyme. ARBs: Angiotensin receptor blocker. bio-ADM: bioactive adrenomedullin. MR-proADM: midregional proadrenomedullin. NT-proBNP: N-terminal pro-B-type natriuretic peptide.

**Supplemental Table 9.** Interaction *p*-values in multivariable models using a cox proportional hazard analysis for predicting all-cause mortality or AHF hospitalizations at 365 days including age, NT-proBNP, creatinine at discharge, and medication at discharge.

|  | **Diuretics** | **ACE inhibitors or ARB** | **Beta blockers** | **Aldosterone antagonists** |
| --- | --- | --- | --- | --- |
| lg NT-proBNP at admission, pg/mL | 0.184 | 0.871 | 0.385 | 0.631 |
| lg NT-proBNP at discharge, pg/mL | 0.007 | 0.258 | 0.135 | 0.028 |

ACE: Angiotensin-converting-enzyme. AHF: Acute heart failure. ARBs: Angiotensin receptor blocker. bio-ADM: bioactive adrenomedullin. MR-proADM: midregional proadrenomedullin. NT-proBNP: N-terminal pro-B-type natriuretic peptide.

**Supplemental Table 10.** Clinical characteristics of patients with bio-ADM concentrations above the median at discharge and without diuretics at discharge who died during the 365-day follow-up (n = 31).

| **PID** | **Age, years** | **Female gender** | **SBP at admission, mmHg** | **SBP at discharge,**  **mmHg** | **bio-ADM at admission, pg/mL** | **bio-ADM at discharge, pg/mL** | **Diuretics at admission** | **ACE inhibitors/ ARBs at admission** | **ACE inhibitors/ ARBs at discharge** | **Creatinine at admission, μmol/L** | **Creatinine at discharge, μmol/L** | **eGFR at discharge,**  **ml/min/1.73m2** | **NT-proBNP at admission, pg/mL** | **NT-proBNP at discharge, pg/mL** | **LVEF** |  |
| --- | --- | --- | --- | --- | --- | --- | --- | --- | --- | --- | --- | --- | --- | --- | --- | --- |
| 76 | 93 | No | 122 | 78 | 71 | 48 | Yes | No | No | 236 | 341 | 16 | 8820.01 | 12771.27 | 33 |  |
| 123 | 88 | No | 135 | 108 | 129 | 188 | Yes | Yes | No | 145 | 131 | 48 | 6341.65 | 11822.22 | 17 |  |
| 184 | 70 | No | 123 | 76 | 38 | 48 | Yes | Yes | No | 83 | 223 | 27 | 733.61 | 849.37 | * |  |
| 209 | 63 | No | 104 | 126 | 103 | 385 | Yes | No | No | 135 | 114 | 60 | 1835.58 | 6253.50 | 58 |  |
| 245 | 73 | Yes | 137 | 68 | 51 | 52 | Yes | Yes | No | 98 | 44 | 129 | 7111.19 | 5848.20 | 41 |  |

| **PID** | **Age, years** | **Female gender** | **SBP at admission, mmHg** | **SBP at discharge,**  **mmHg** | **bio-ADM at admission, pg/mL** | **bio-ADM at discharge, pg/mL** | **Diuretics at admission** | **ACE inhibitors/ ARBs at admission** | **ACE inhibitors/ ARBs at discharge** | **Creatinine at admission, μmol/L** | **Creatinine at discharge, μmol/L** | **eGFR at**  **discharge,**  **ml/min/1.73m^2^** | **NT-proBNP at admission, pg/mL** | **NT-proBNP at discharge, pg/mL** | **LVEF** |  |  |  |  |  |  |  |  |  |  |
| --- | --- | --- | --- | --- | --- | --- | --- | --- | --- | --- | --- | --- | --- | --- | --- | --- | --- | --- | --- | --- | --- | --- | --- | --- | --- |
| 296 | 89 | No | 120 | 110 | 30 | 77 | Yes | Yes | No | 113 | 123 | 51 | 968 | 2143 | * |  |  |  |  |  |  |  |  |  |  |
| 327 | 88 | No | 99 | * | 95 | 99 | Yes | No | No | 148 | 143 | 43 | 10537 | 10323 | * |  |  |  |  |  |  |  |  |  |  |
| 374 | 64 | No | 106 | * | 107 | 47 | Yes | Yes | No | 93 | 103 | 67 | 1278 | 1005 | 39 |  |  |  |  |  |  |  |  |  |  |
| 386 | 92 | Yes | 184 | * | 31 | 71 | Yes | Yes | No | 118 | 283 | 14 | 2057 | 2913 | 47 |  |  |  |  |  |  |  |  |  |  |
| 395 | 78 | No | 84 | 145 | 127 | 163 | Yes | Yes | No | 185 | * | * | 4816 | 4328 | 65 |  |  |  |  |  |  |  |  |  |  |
| 446 | 75 | No | 138 | 135 | 54 | 84 | Yes | No | No | 368 | 348 | 16 | 8607 | 8902 | 48 |  |  |  |  |  |  |  |  |  |  |
| **PID** | **Age, years** | **Female gender** | **SBP at admission, mmHg** | **SBP at discharge,**  **mmHg** | **bio-ADM at admission, pg/mL** | **bio-ADM at discharge, pg/mL** | **Diuretics at admission** | **ACE inhibitors/ ARBs at admission** | **ACE inhibitors/ ARBs at discharge** | **Creatinine at admission, μmol/L** | **Creatinine at discharge, μmol/L** | **eGFR at**  **discharge,**  **ml/min/1.73m^2^** | **NT-proBNP at admission, pg/mL** | **NT-proBNP at discharge, pg/mL** | **LVEF** |  |  |  |  |  |  |  |  |  |  |
| 516 | 78 | No | 155 | 115 | 217 | 210 | Yes | Yes | No | 151 | 291 | 19 | 7739 | 6934 | 24 |  |  |  |  |  |  |  |  |  |  |
| 525 | 82 | Yes | 136 | 68 | 62 | 53 | No | Yes | No | 110 | 117 | 41 | 5925 | * | 34 |  |  |  |  |  |  |  |  |  |  |
| 542 | 64 | No | 128 | * | 1773 | 734 | No | No | No | 135 | 83 | 86 | 2319 | 1855 | 15 |  |  |  |  |  |  |  |  |  |  |
| 545 | 74 | No | 118 | * | 572 | 539 | Yes | No | No | 234 | 318 | 18 | 2422 | 2713 | 41 |  |  |  |  |  |  |  |  |  |  |
| 546 | 92 | No | * | 86 | 92 | 238 | Yes | No | No | 107 | 133 | 34 | 1845 | 1828 | 24 |  |  |  |  |  |  |  |  |  |  |
| 554 | 98 | No | 110 | * | 176 | 182 | Yes | No | No | 176 | 292 | 18 | 2598 | 2753 | 34 |  |  |  |  |  |  |  |  |  |  |
| **PID** | **Age, years** | **Female gender** | **SBP at admission, mmHg** | **SBP at discharge,**  **mmHg** | **bio-ADM at admission, pg/mL** | **bio-ADM at discharge, pg/mL** | **Diuretics at admission** | **ACE inhibitors/ ARBs at admission** | **ACE inhibitors/ ARBs at discharge** | **Creatinine at admission, μmol/L** | **Creatinine at discharge, μmol/L** | **eGFR at**  **discharge,**  **ml/min/1.73m^2^** | **NT-proBNP at admission, pg/mL** | **NT-proBNP at discharge, pg/mL** | **LVEF** |  |  |  |  |  |  |  |  |  |  |
| 561 | 65 | No | 113 | 121 | 45 | 50 | Yes | Yes | No | 83 | 143 | 46 | 946 | 141 | 32 |  |  |  |  |  |  |  |  |  |  |
| 568 | 78 | No | 150 | 110 | 132 | 95 | No | Yes | No | 201 | * | * | 2010 | 1848 | * |  |  |  |  |  |  |  |  |  |  |
| 614 | 89 | No | 158 | 130 | 195 | 166 | Yes | Yes | No | 265 | 403 | 13 | 2082 | 1613 | 47 |  |  |  |  |  |  |  |  |  |  |
| 623 | 89 | Yes | 135 | 89 | 158 | 98 | Yes | Yes | No | 147 | 147 | 31 | 2334 | 2756 | * |  |  |  |  |  |  |  |  |  |  |
| 624 | 86 | Yes | 171 | 164 | 108 | 154 | No | Yes | No | 97 | 155 | 29 | 2997 | 3747 | * |  |  |  |  |  |  |  |  |  |  |
| 806 | 77 | No | 110 | 90 | 87 | 130 | Yes | Yes | No | 95 | 104 | 64 | 1971 | 1785 | 20 |  |  |  |  |  |  |  |  |  |  |
| **PID** | **Age, years** | **Female gender** | **SBP at admission, mmHg** | **SBP at discharge,**  **mmHg** | **bio-ADM at admission, pg/mL** | **bio-ADM at discharge, pg/mL** | **Diuretics at admission** | **ACE inhibitors/ ARBs at admission** | **ACE inhibitors/ ARBs at discharge** | **Creatinine at admission, μmol/L** | **Creatinine at discharge, μmol/L** | **eGFR at**  **discharge,**  **ml/min/1.73m^2^** | **NT-proBNP at admission, pg/mL** | **NT-proBNP at discharge, pg/mL** | **LVEF** |  |  |  |  |  |  |  |  |  |  |
| 844 | 81 | Yes | 130 | 67 | 86 | 154 | Yes | Yes | No | 124 | 277 | 15 | 3333 | 2422 | 64 |  |  |  |  |  |  |  |  |  |  |
| 860 | 85 | No | 108 | 75 | 88 | 80 | Yes | Yes | No | 146 | 199 | 30 | 1607 | 1525 | 25 |  |  |  |  |  |  |  |  |  |  |
| 952 | 87 | No | 110 | 82 | 68 | 149 | Yes | No | No | 265 | 277 | 20 | 6334 | 7025 | 9 |  |  |  |  |  |  |  |  |  |  |
| 1028 | 59 | Yes | 117 | 56 | 249 | 96 | Yes | Yes | No | 274 | 398 | 11 | 2656 | 1654 | 23 |  |  |  |  |  |  |  |  |  |  |
| 1120 | 70 | No | 108 | 81 | 62 | 242 | Yes | Yes | No | 126 | 136 | 48 | 2313 | 1118 | 15 |  |  |  |  |  |  |  |  |  |  |
| 1159 | 68 | No | 113 | * | 130 | 72 | Yes | Yes | No | 122 | 136 | 48 | 3219 | 1710 | 27 |  |  |  |  |  |  |  |  |  |  |
| **PID** | **Age, years** | **Female gender** | **SBP at admission, mmHg** | **SBP at discharge,**  **mmHg** | **bio-ADM at admission, pg/mL** | **bio-ADM at discharge, pg/mL** | **Diuretics at admission** | **ACE inhibitors/ ARBs at admission** | **ACE inhibitors/ ARBs at discharge** | **Creatinine at admission, μmol/L** | **Creatinine at discharge, μmol/L** | **eGFR at**  **discharge,**  **ml/min/1.73m^2^** | **NT-proBNP at admission, pg/mL** | **NT-proBNP at discharge, pg/mL** | **LVEF** |  |  |  |  |  |  |  |  |  |  |
| 1174 | 83 | No | 130 | 120 | 107 | 41 | No | Yes | No | 114 | 108 | 60 | 1936 | 1711 | 20 |  |  |  |  |  |  |  |  |  |  |
| 1819 | 75 | No | 87 | 93 | 206 | 87 | Yes | Yes | Yes | 223 | 92 | 74 | 9076 | 7995 | 20 |  |  |  |  |  |  |  |  |  |  |

* Data not available.

ACE: Angiotensin-converting-enzyme. ARBs: Angiotensin receptor blocker. bio-ADM: bioactive adrenomedullin. eGFR: estimated glomerular filtration rate using the chronic kidney disease epidemiology collaboration formula. LVEF: Left ventricular ejection fraction. NT-proBNP: N-terminal pro-B-type natriuretic peptide. PID: patient’s identification number. SBP: systolic blood pressure.

**Supplemental Table 11A.** Multivariable Cox proportional hazards model for mortality at 365 days including bio-ADM (n = 326).

| **Variable** | **Hazard ratio** | **95% CI** | ***p-value*** |
| --- | --- | --- | --- |
| Age (years) | 1.047 | 1.017-1.077 | 0.002 |
| Beta blockers at baseline |  |  | 0.062 |
| lg BUN (mmol/L) |  |  | 0.132 |
| lg Haemoglobin (g/L) |  |  | 0.629 |
| lg NT-proBNP (ng/L) | 3.216 | 1.810-5.714 | < 0.001 |
| Haemoconcentration* |  |  | 0.093 |
| lg bioADM* (ng/L) | 3.427 | 1.286-9.133 | 0.014 |

*at discharge

bio-ADM: bioactive adrenomedullin. BUN: Blood urea nitrogen. CI: Confidence interval. NT-proBNP: N-terminal pro-B-type natriuretic peptide.

**Supplemental Table 11B.** Multivariable Cox proportional hazards model for mortality at 365 days including MR-proADM (n = 204).

| **Variable** | **Hazard ratio** | **95% CI** | ***p-value*** |
| --- | --- | --- | --- |
| Age (years) | 1.042 | 1.007-1.078 | 0.017 |
| Beta blockers at baseline |  |  | 0.267 |
| lg BUN (mmol/L) |  |  | 0.553 |
| lg Haemoglobin (g/L) |  |  | 0.580 |
| lg NT-proBNP (ng/L) | 3.975 | 1.819-8.690 | 0.001 |
| Haemoconcentration* |  |  | 0.747 |
| lg MR-proADM* (ng/L) | 87.612 | 4.291-1,788.949 | 0.004 |

* at discharge

BUN: Blood urea nitrogen. CI: Confidence interval. NT-proBNP: N-terminal pro-B-type natriuretic peptide. MR-proADM: midregional proadrenomedullin. NT-proBNP: N-terminal pro-B-type natriuretic peptide.

**Supplemental Table 12.** Patient’s characteristics according to survival status at 365 days after excluding patients receiving palliative care.

|  | **Dead at 365 Days (n = 504)** | **Alive at 365 Days (n = 1,372)** | ***p*-value** |
| --- | --- | --- | --- |
| **Demographics** |  |  |  |
| Age, years | 81.5 (75.0-87.0) | 76.5 (67.0-83.0) | < 0.001 |
| Female gender, % | 37.0 | 38.2 | 0.631 |
| BMI, kg/m^2^ | 24.9 (21.8-28.4) | 28.1 (24.6-32.8) | < 0.001 |
| **Clinical parameters at ED** |  |  |  |
| SBP, mmHg | 126 (110-146) | 137 (120-155) | < 0.001 |
| HR, beats/min | 85 (72-101) | 87 (72-105) | 0.125 |
| LV Ejection Fraction, % | 35 (24-49) | 40 (29-54) | < 0.001 |
| LVEDD, cm | 5.3 (4.6-6.1) | 5.2 (4.6-5.9) | 0.317 |
| LVESD, cm | 4.20 (3.4-5.2) | 4.20 (3.3-5.0) | 0.387 |
| **Medical history** |  |  |  |
| CKD, % | 44.8 | 28.9 | < 0.001 |
| Hypertension, % | 67.9 | 70.4 | 0.313 |
| Dyslipidaemia, % | 41.2 | 42.4 | 0.687 |
| Stroke or TIA, % | 21.3 | 14.3 | 0.001 |
| Current or ex-smoker, % | 57.9 | 54.6 | 0.266 |
| PAD, % | 13.3 | 9.7 | 0.046 |
| Atrial fibrillation, % | 40.5 | 43.1 | 0.374 |
| COPD, % | 17.7 | 15.5 | 0.326 |
| Diabetes, % | 31.7 | 32.1 | 0923 |
| **Medication at presentation** |  |  |  |
| ACE inhibitors or ARB, % | 58.8 | 62.3 | 0.216 |
| Beta blockers, % | 52.2 | 54.1 | 0.526 |
| Aldosterone antagonists, % | 16.9 | 11.7 | 0.008 |
| Loop diuretics, % | 77.0 | 61.0 | < 0.001 |
|  |  |  |  |
|  |  |  |  |
|  |  |  |  |
|  |  |  |  |

|  | **Dead at 365 Days (n = 504)** | **Alive at 365 Days (n = 1,372)** | | ***p*-value** |  |
| --- | --- | --- | --- | --- | --- |
| **Medication at discharge** | |  |  | | |
| ACE inhibitors or ARB, % | 52.6 | 76.3 | < 0.001 | | |
| Beta blockers, % | 46.7 | 68.3 | < 0.001 | | |
| Aldosterone antagonists, % | 26.2 | 33.0 | 0.006 | | |
| Loop diuretics, % | 77.4 | 88.3 | < 0.001 | | |
| **Laboratory parameters at admission** | |  | |  |  |
| Haemoglobin, g/L | 120 (108-132) | 127 (113-139) | | < 0.001 |  |
| Sodium, mmol/L | 138 (134-141) | 139 (136-141) | | < 0.001 |  |
| Potassium, mmol/L | 4.4 (3.9-4.8) | 4.2 (3.9-4.6) | | 0.004 |  |
| Creatinine, μmol/L | 132 (100.0-180.0) | 105.0 (83.0-134.0) | | < 0.001 |  |
| Urea, mmol/L | 12.3 (8.7-17.8) | 8.4 (6.2-11.7) | | < 0.001 |  |
| NT-proBNP, pg/mL | 4,745 (2,381-9,131) | 2,629 (1,254-5,342) | | < 0.001 |  |
| BNP, pg/mL | 1,897 (810-3,036) | 1120 (605-2,128) | | 0.009 |  |
| Bio-ADM, pg/mL | 56.9 (35.2-94.3) | 41.5 (28.8-62.0) | | < 0.001 |  |
| MR-proADM, nmol/L | 2.24 (1.68- 3.04) | 1.50 (1.14-2.14) | | < 0.001 |  |
| **Laboratory parameters at discharge** | |  | |  |  |
| Creatinine, μmol/L | 132.0 (100.0-191.0) | 107.0 (85.0-135.0) | | < 0.001 |  |
| NT-proBNP, pg/mL | 3,327 (1,683-8,438) | 1,659 (686-3,417) | | < 0.001 |  |
| Bio-ADM, pg/mL | 43.9 (28.7-71.2) | 34.4 (25.2-51.7) | | < 0.001 |  |
| MR-proADM, nmol/L | 1.77 (1.44-2.55) | 1.29 (0.99-1.83) | | < 0.001 |  |

ACE: Angiotensin-converting-enzyme. ARBs: Angiotensin receptor blocker. BMI: Body mass index. bio-ADM: bioactive adrenomedullin. CAD: Coronary artery disease. CCB: Calcium channel blockers. COPD: Chronic obstructive pulmonary disease. CKD: Chronic kidney disease. ED: Emergency department. HR: Heart rate. MR-proADM: midregional proadrenomedullin. LV: Left ventricle. PAD: Peripheral artery disease. SBP: Systolic blood pressure.

**Supplemental Table 13.** Interaction *p*-values in bivariable models using a cox proportional hazard analysis for predicting all-cause mortality at 365 days including one biomarker and medication at discharge after excluding patients receiving palliative care.

|  | **Diuretics** | **ACE inhibitors or ARB** | **Beta blockers** | **Aldosterone antagonists** |
| --- | --- | --- | --- | --- |
| lg bio-ADM at admission, pg/mL | **<0.001** | **0.003** | 0.107 | 0.015 |
| lg bio-ADM at discharge, pg/mL | **< 0.001** | 0.051 | 0.809 | 0.350 |
| lg MR-proADM at admission, nmol/L | 0.068 | 0.209 | 0.113 | 0.562 |
| lg MR-proADM at discharge, nmol/L | 0.071 | 0.518 | 0.305 | 0.201 |

ACE: Angiotensin-converting-enzyme. ARBs: Angiotensin receptor blocker. bio-ADM: bioactive adrenomedullin. MR-proADM: midregional proadrenomedullin.

**Supplemental Table 14.** Interaction *p*-values in multivariable models using a cox proportional hazard analysis for predicting all-cause mortality at 365 days including age, bio-ADM or MR-proADM, NT-proBNP at discharge, creatinine at discharge, and medication at discharge after excluding patients receiving palliative care.

|  | **Diuretics** | **ACE inhibitors or ARB** | **Beta blockers** | **Aldosterone antagonists** |
| --- | --- | --- | --- | --- |
| lg bio-ADM at admission, pg/mL | **< 0.001** | 0.108 | 0.618 | 0.590 |
| lg bio-ADM at discharge, pg/mL | **0.002** | 0.032 | 0.938 | 0.625 |
| lg MR-proADM at admission, nmol/L | 0.600 | 0.419 | 0.819 | 0.121 |
| lg MR-proADM at discharge, nmol/L | 0.146 | 0.749 | 0.581 | 0.914 |

ACE: Angiotensin-converting-enzyme. ARBs: Angiotensin receptor blocker. bio-ADM: bioactive adrenomedullin. MR-proADM: midregional proadrenomedullin. NT-proBNP: N-terminal pro-B-type natriuretic peptide.

**Supplemental Table 15.** Interaction *p*-values in bivariable models using a cox proportional hazard analysis for predicting all-cause mortality or AHF hospitalizations at 365 days including one biomarker, and medication at discharge after excluding patients receiving palliative care.

|  | **Diuretics** | **ACE inhibitors or ARB** | **Beta blockers** | **Aldosterone antagonists** |
| --- | --- | --- | --- | --- |
| lg bio-ADM at admission, pg/mL | **< 0.001** | 0.009 | 0.305 | 0.287 |
| lg bio-ADM at discharge, pg/mL | **< 0.001** | 0.066 | 0.931 | 0.777 |
| lg MR-proADM at admission, nmol/L | 0.022 | 0.049 | 0.023 | 0.493 |
| lg MR-proADM at discharge, nmol/L | 0.026 | 0.048 | 0.678 | 0.575 |

ACE: Angiotensin-converting-enzyme. AHF: Acute heart failure. ARBs: Angiotensin receptor blocker. bio-ADM: bioactive adrenomedullin. MR-proADM: midregional proadrenomedullin

**Supplemental Table 16.** Interaction *p*-values in multivariable models using a cox proportional hazard analysis or predicting all-cause mortality or AHF hospitalizations at 365 days including age, bio-ADM or MR-proADM, NT-proBNP at discharge, creatinine at discharge, and medication at discharge after excluding patients receiving palliative care.

|  | **Diuretics** | **ACE inhibitors or ARB** | **Beta blockers** | **Aldosterone antagonists** |
| --- | --- | --- | --- | --- |
| lg bio-ADM at admission, pg/mL | **0.001** | 0.207 | 0.513 | 0.595 |
| lg bio-ADM at discharge, pg/mL | **0.001** | 0.053 | 0.608 | 0.994 |
| lg MR-proADM at admission, nmol/L | 0.345 | 0.322 | 0.340 | 0.053 |
| lg MR-proADM at discharge, nmol/L | 0.067 | 0.153 | 0.696 | 0.950 |

ACE: Angiotensin-converting-enzyme. AHF: Acute heart failure. ARBs: Angiotensin receptor blocker. bio-ADM: bioactive adrenomedullin. MR-proADM: midregional proadrenomedullin. NT-proBNP: N-terminal pro-B-type natriuretic peptide.

**Supplemental Table 17.** Interaction *p*-values in multivariable models using a cox proportional hazard analysis for predicting all-cause mortality at 365 days including age, NT-proBNP, creatinine at discharge, and medication at discharge after excluding patients receiving palliative care.

|  | **Diuretics** | **ACE inhibitors or ARB** | **Beta blockers** | **Aldosterone antagonists** |
| --- | --- | --- | --- | --- |
| lg NT-proBNP at admission, pg/mL | 0.375 | 0.944 | 0.292 | 0.810 |
| lg NT-proBNP at discharge, pg/mL | 0.023 | 0.254 | 0.249 | 0.537 |

ACE: Angiotensin-converting-enzyme. ARBs: Angiotensin receptor blocker. NT-proBNP: N-terminal pro-B-type natriuretic peptide.

**Supplemental Table 18.** Interaction *p*-values in multivariable models using a cox proportional hazard analysis for predicting all-cause mortality or AHF hospitalizations at 365 days including age, NT-proBNP, creatinine at discharge, and medication at discharge after excluding patients receiving palliative care.

|  | **Diuretics** | **ACE inhibitors or ARB** | **Beta blockers** | **Aldosterone antagonists** |
| --- | --- | --- | --- | --- |
| lg NT-proBNP at admission, pg/mL | 0.199 | 0.799 | 0.406 | 0.581 |
| lg NT-proBNP at discharge, pg/mL | 0.030 | 0.249 | 0.119 | 0.028 |

ACE: Angiotensin-converting-enzyme. AHF: Acute heart failure. ARBs: Angiotensin receptor blocker. NT-proBNP: N-terminal pro-B-type natriuretic peptide.

**Figure legends**

**Supplemental Figure 1.** Patient flow diagram.

**Supplemental Figure 2.** Patients’ all-cause mortality according to study site (n = 1,886).

**Supplemental Figure 3.** Patients’ all-cause mortality or AHF hospitalizations according to study site (n = 1,671).

**Supplemental Figure 4.** Time dependent ROC curves describing the prognostic performance of admission bio-ADM and MR-proADM to predict all-cause mortality or AHF rehospitalisations during 365-day follow-up (n = 747).

**Supplemental Figures 5A and B.** All-cause mortality or AHF rehospitalisations stratified according to bio-ADM concentration and the use of diuretics at discharge: A) Values at admission (n = 1,629). B) Values at discharge (n = 997).

**Supplemental Figures 6A and B.** All-cause mortality or AHF rehospitalisations stratified according to MR-proADM concentration and the use of diuretics at discharge: A) Values at admission (n = 721). B) Values at discharge (n = 438).

**Supplemental Figure 7.** Mortality according to bio-ADM and MR-proADM concentrations at presentation and acute ward discharge. A) Values at admission, B) Values at discharge. Bio-ADM levels were available for 1,886 patients at admission and 1,001 patients at discharge. MR-proADM levels were available for 764 patients at admission and 440 patients at discharge.

**Supplemental Figure 8.** Time dependent ROC curves describing the prognostic performance of admission bio-ADM, the OPTIMIZE-HF risk score, and their combination to predict all-cause mortality during 365-day follow-up (n = 1,706).

**Supplemental Figure 9.** Time dependent ROC curves describing the prognostic performance of admission MR-proADM, the OPTIMIZE-HF risk score, and their combination to predict all-cause mortality during 365-day follow-up (n = 725).

**Supplemental Figure 10.** All-cause mortality according to study site after excluding patients receiving palliative care (n = 1,876).

**Supplemental Figure 11.** All-cause mortality or AHF rehospitalisations according to study site after excluding patients receiving palliative care (n = 1,661).

**Supplemental Figure 12.** Mortality stratified according to bio-ADM and MR-proADM concentration and the use of diuretics at discharge after excluding patients receiving palliative care: A) bio-ADM at presentation (n = 1,834); B) bio-ADM at discharge (n = 987).

**Supplemental Figure 13.** All-cause mortality or AHF rehospitalisations according bio-ADM concentration and the use of diuretics at discharge after excluding patients receiving palliative care: A) Values at admission (n = 1,619). B) Values at discharge (n = 987).

**Supplemental Figure 14**. Time dependent ROC curves describing the performance of bio-ADM and MR-proADM to predict all-cause mortality during 365-day follow-up (n = 758) after excluding patients receiving palliative care

**Supplemental Figure 15.** Time dependent ROC curves describing the prognostic performance of bio-ADM and MR-proADM to predict all-cause mortality or AHF rehospitalisations during 365-day follow-up after excluding patients receiving palliative care (n=741).

**Supplemental Figure 16.** Mortality according to bio-ADM and MR-proADM concentrations at presentation and acute ward discharge after excluding patients receiving palliative care. A) Values at admission, B) Values at discharge. Bio-ADM levels were available for 1,876 patients at admission and 991 patients at discharge. MR-proADM levels were available for 758 patients at admission and 435 patients at discharge.

# **Figures**

**Supplemental Figure 1.** Patient flow diagram.

1,001 patients with bio-ADM measurements at discharge

764 patients with MR-proADM measurements at admission

440 patients with MR-proADM measurements at discharge

bio-ADM: bioactive adrenomedullin. MR-proADM: midregional proadrenomedullin.

2,069 patients enrolled

183 patients without bio-ADM measurements at admission

1,886 patients included

in this analysis

**Supplemental Figure 2.** Patients’ all-cause mortality according to study site (n = 1,886).

**
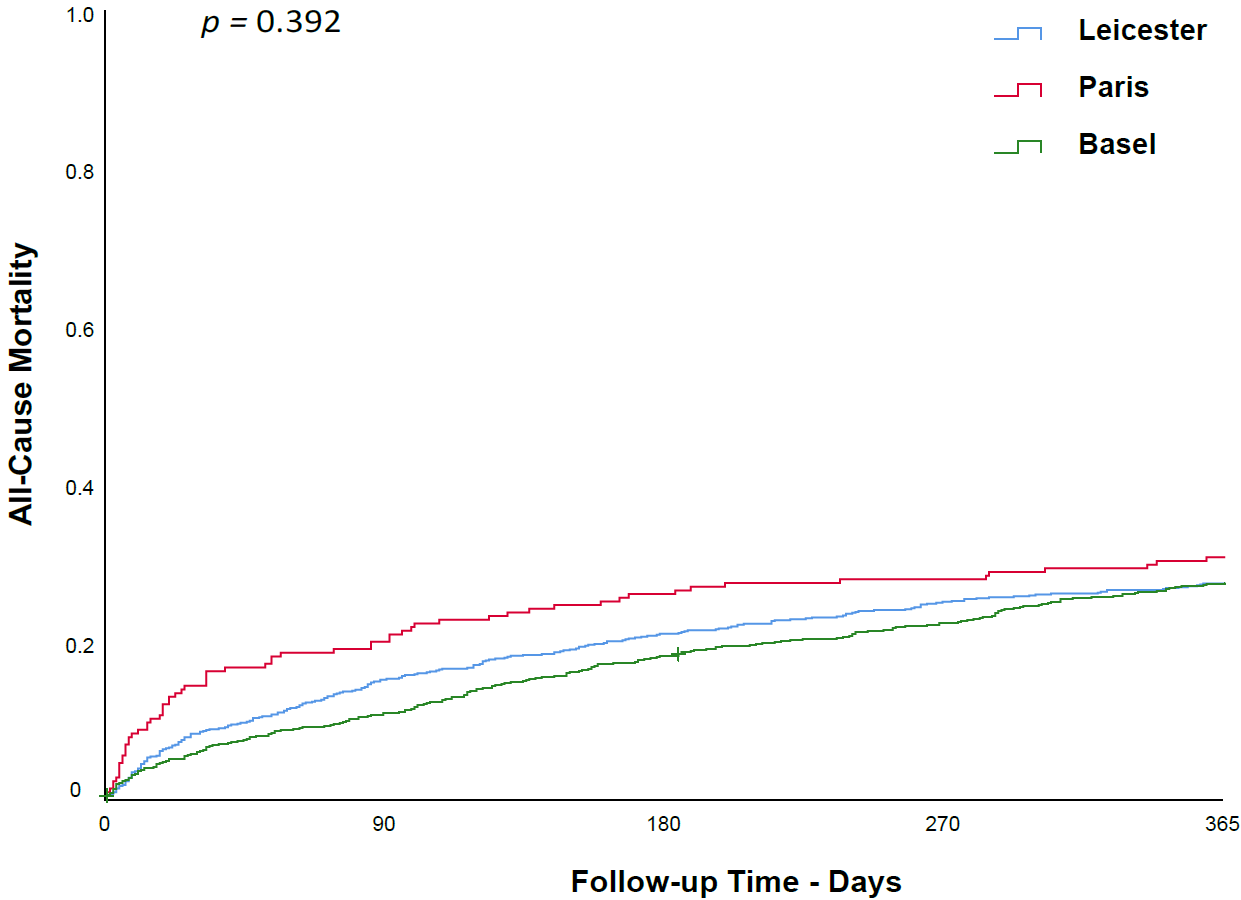
**

**Supplemental Figure 3.** Patients’ all-cause mortality or AHF rehospitalisations according to study site (n = 1,671).


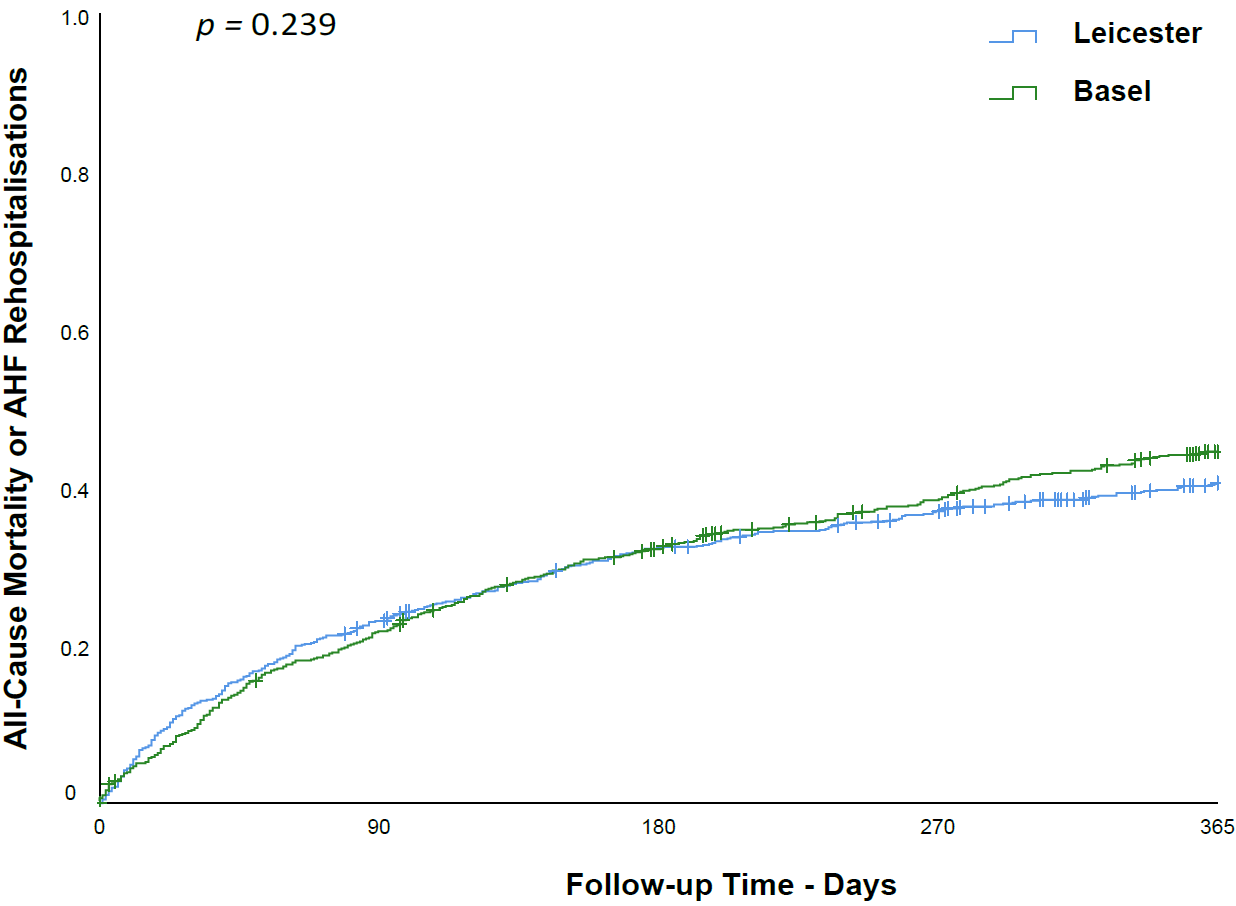


AHF: Acute heart failure.

**Supplemental Figure 4.** All-cause mortality and AHF hospitalizations stratified according to bio-ADM concentration and the use of diuretics at discharge: A) Values at admission (n = 1,629). B) Values at discharge (n = 997).


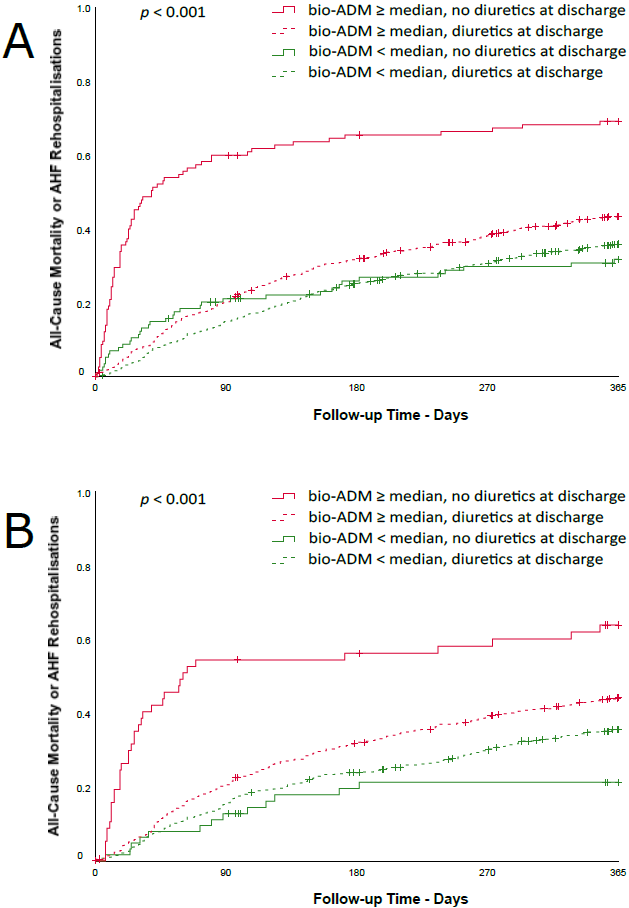


AHF: Acute heart failure. bio-ADM: bioactive adrenomedullin.

**Supplemental Figure 5.** All-cause mortality and AHF hospitalizations stratified according to MR-proADM concentration and the use of diuretics at discharge: A) Values at admission (n = 721). B) Values at discharge (n = 438).


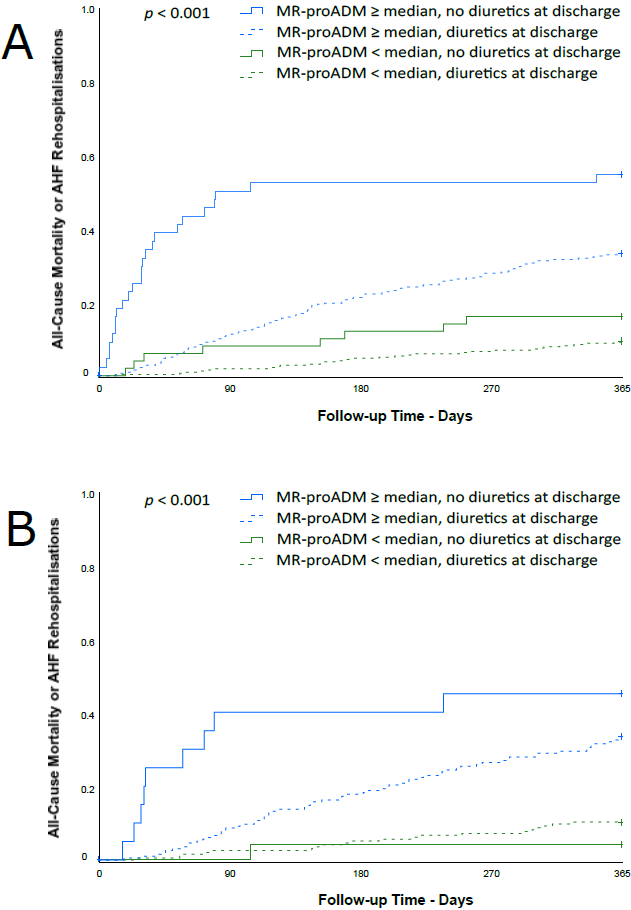


AHF: Acute heart failure. MR-proADM: midregional proadrenomedullin.

**Supplemental Figure 6.** Time dependent ROC curves describing the prognostic performance of admission bio-ADM and MR-proADM to predict all-cause mortality or AHF hospitalizations during 365-day follow-up (n= 747).


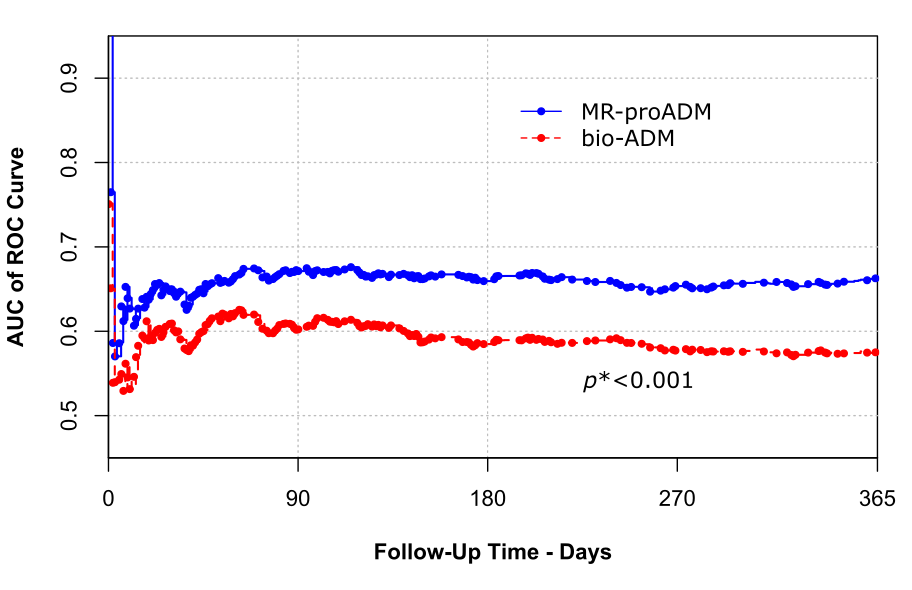


* at 90, 180, and 365 days

AHF: Acute heart failure. AUC of ROC curve: area under the time dependent receiver operating characteristic curve. bio-ADM: bioactive adrenomedullin. MR-proADM: midregional proadrenomedullin.

**Supplemental Figure 7.** Mortality according to bio-ADM and MR-proADM concentrations at presentation and acute ward discharge. A Values at admission, B Values at discharge. Bio-ADM levels were available for 1,886 patients at admission and 1,001 patients at acute ward discharge. MR-proADM levels were available for 764 patients at admission and 440 patients at acute ward discharge.

**
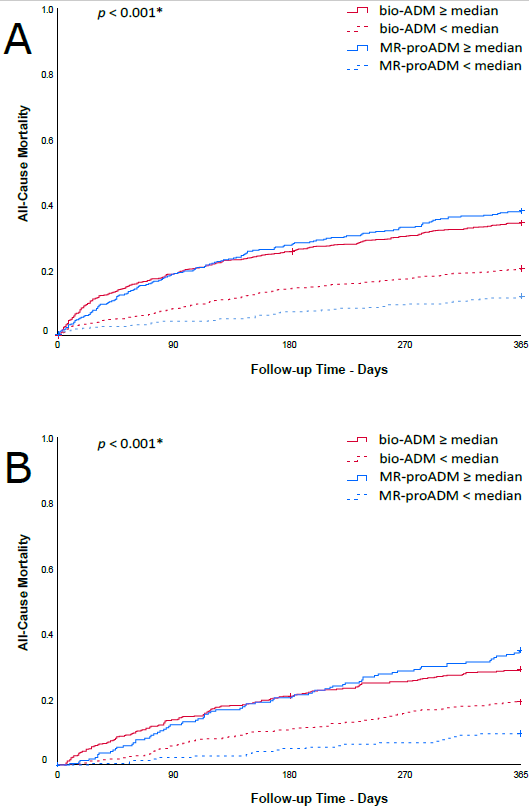
**

***** for bio-ADM and also MR-proADM

bio-ADM: bioactive adrenomedullin. MR-proADM: midregional proadrenomedullin.

**Supplemental Figure 8.** Time dependent ROC curves describing the prognostic performance of admission bio-ADM, the OPTIMIZE-HF risk score, and their combination* to predict all-cause mortality during 365-day follow-up (n = 1,706).


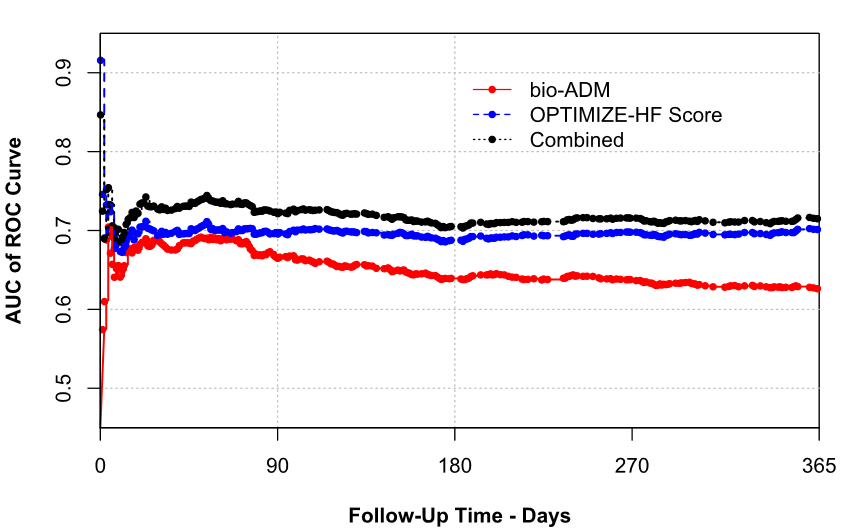


* There was a significant improvement of the prognostic accuracy of the OPTIMIZE-HF risk score when combining it with bio-ADM throughout 90 days follow-up (*p*-value < 0.001 at 90 as well as at 60 days follow-up).

AUC of ROC curve: area under the time dependent receiver operating characteristic curve. bio-ADM: bioactive adrenomedullin.

**Supplemental Figure 9.** Time dependent ROC curves describing the prognostic performance of admission MR-proADM, the OPTIMIZE-HF risk score, and their combination* to predict all-cause mortality during 365-day follow-up (n = 725).


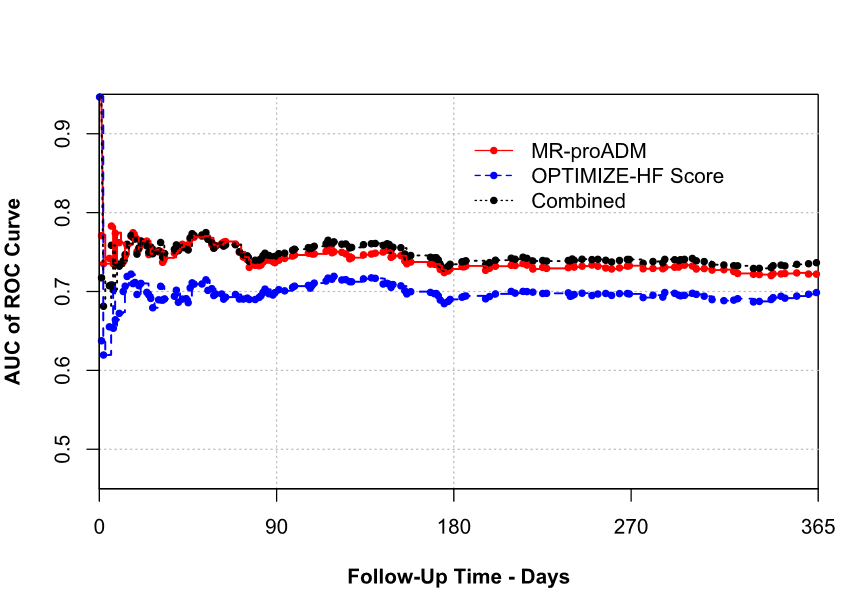


* There was a significant improvement of the prognostic accuracy of the OPTIMIZE-HF risk score when combining it with MR-proADM throughout 90 days follow-up (*p*-value = 0.006 at 90 days and < 0.001 at 60 follow-up).

AUC of ROC curve: area under the time dependent receiver operating characteristic curve. MR-proADM: midregional proadrenomedullin.

**Supplemental Figure 10.** All-cause mortality according to study site after excluding patients receiving palliative care (n = 1,876).


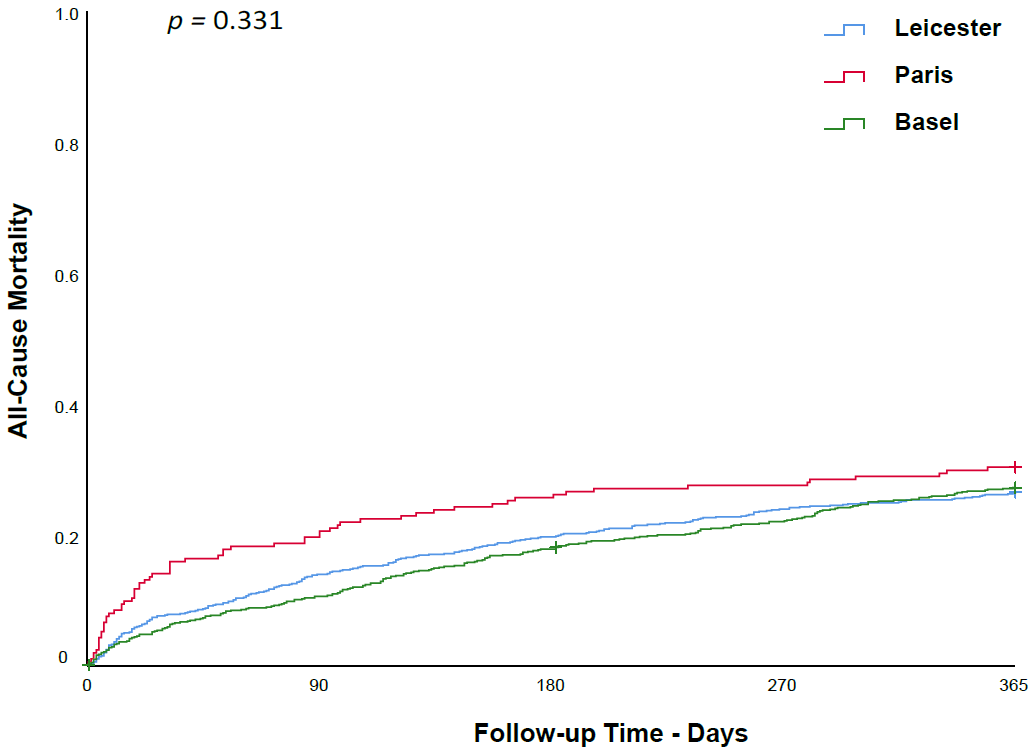


**Supplemental Figure 11.** All-cause mortality and/or AHF hospitalizations according to study site after excluding patients receiving palliative care (n = 1,661).


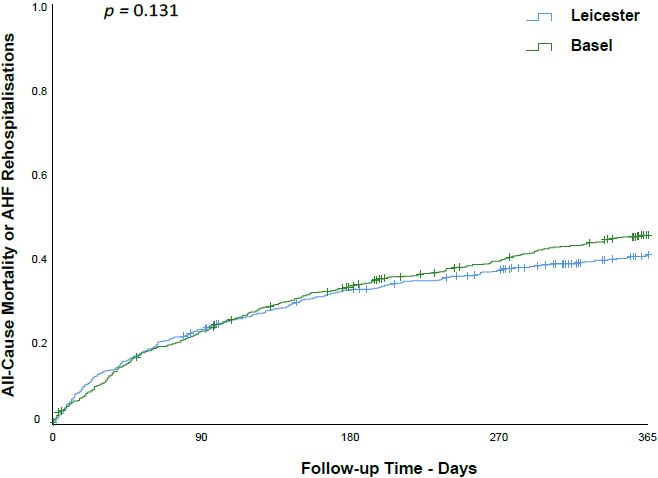


AHF: Acute heart failure.

**Supplemental Figure 12.** All-cause mortality stratified according to bio-ADM concentration and the use of diuretics at discharge after excluding patients receiving palliative care: A) bio-ADM at presentation (n = 1,834); B) MR-proADM at presentation (n = 732); C) bio-ADM at discharge (n = 987); D) MR-proADM at discharge (n = 433).


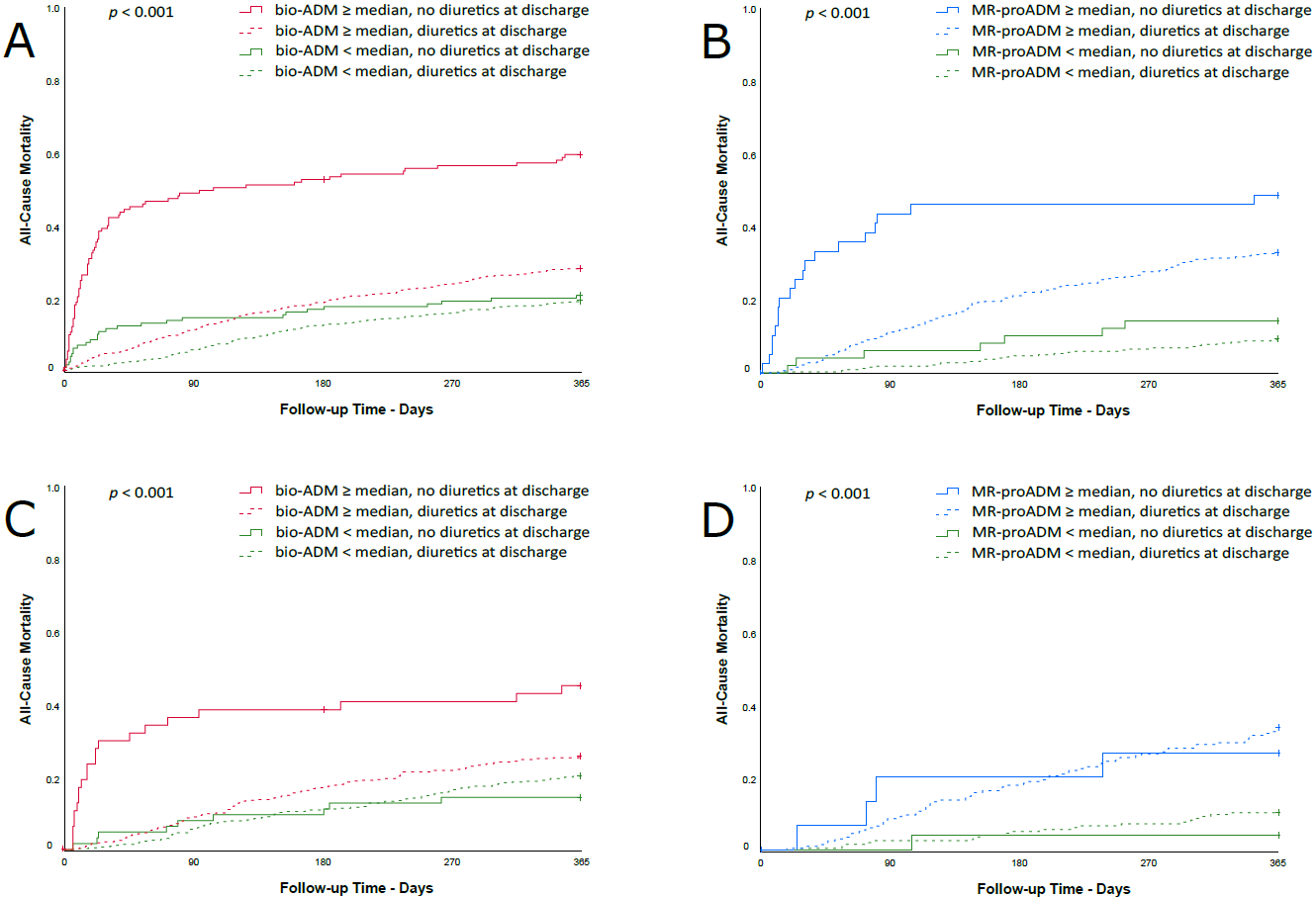


bio-ADM: bioactive adrenomedullin. MR-proADM: midregional proadrenomedullin.

**Supplemental Figure 13.** All-cause mortality and AHF hospitalizations stratified according to bio-ADM concentration and the use of diuretics at discharge after excluding patients receiving palliative care: A) bio-ADM at presentation (n = 1,619); B) MR-proADM at presentation (n = 715); C) bio-ADM at discharge (n = 987); D) MR-proADM at discharge (n = 433).


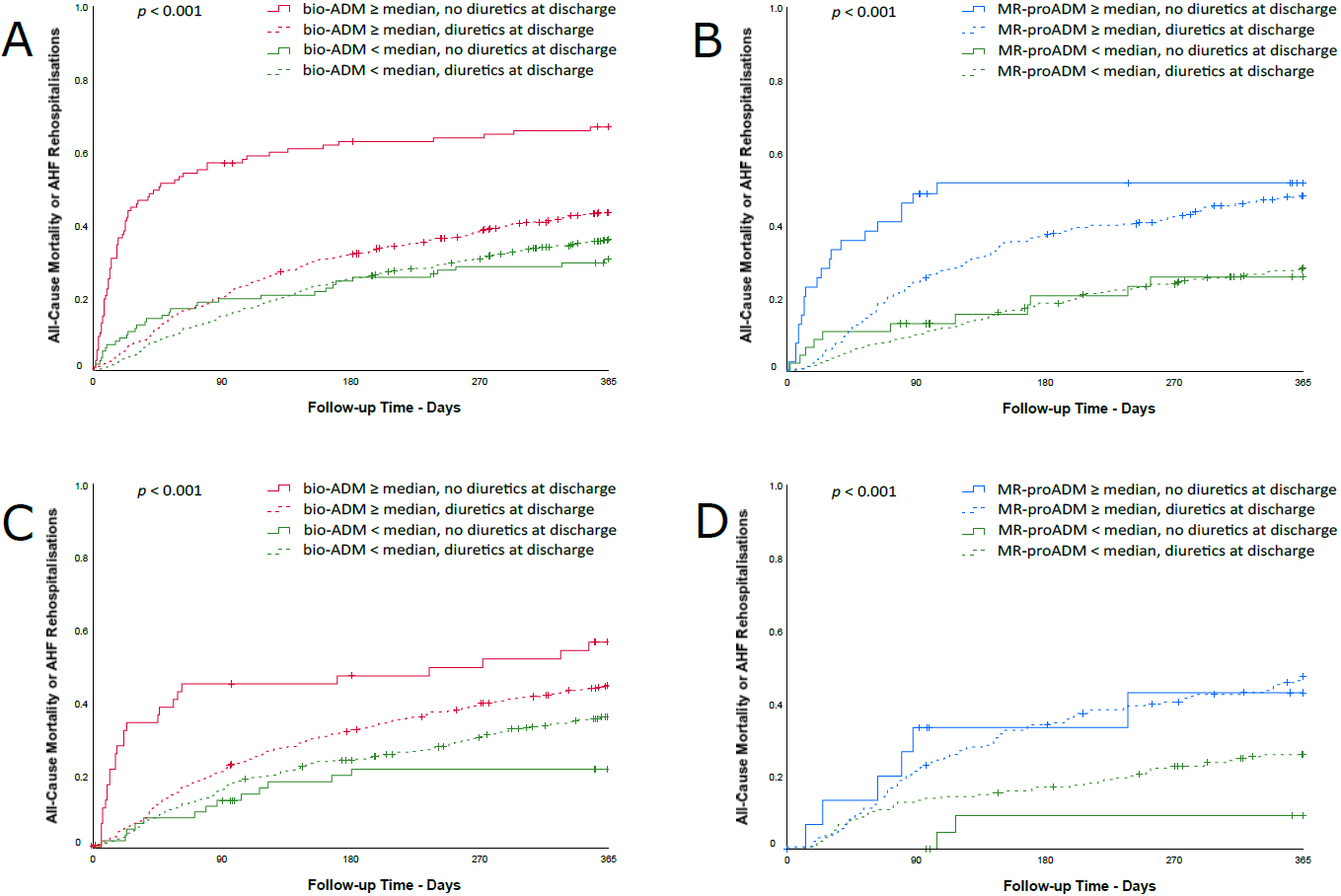


AHF: Acute heart failure. bio-ADM: bioactive adrenomedullin. MR-proADM: midregional proadrenomedullin.

**Supplemental Figure 14.** Time dependent ROC curves describing the prognostic performance of admission bio-ADM and MR-proADM to predict all-cause mortality during 365-day follow-up (n = 758) after excluding patients receiving palliative care.


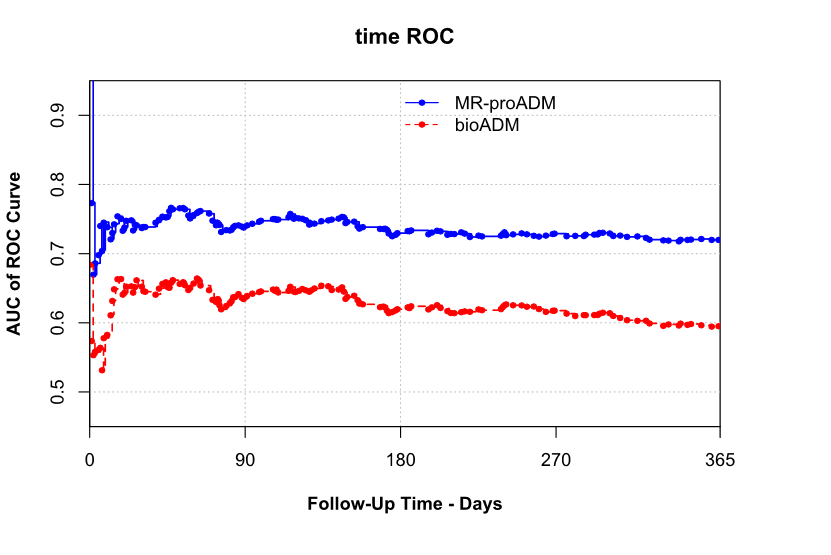


AUC of ROC curve: area under the time dependent receiver operating characteristic curve. bio-ADM: bioactive adrenomedullin. MR-proADM: midregional proadrenomedullin.

**Supplemental Figure 15.** Time dependent ROC curves describing the prognostic performance of admission bio-ADM and MR-proADM to predict all-cause mortality or AHF hospitalizations during 365-day follow-up after excluding patients receiving palliative care (n=741).


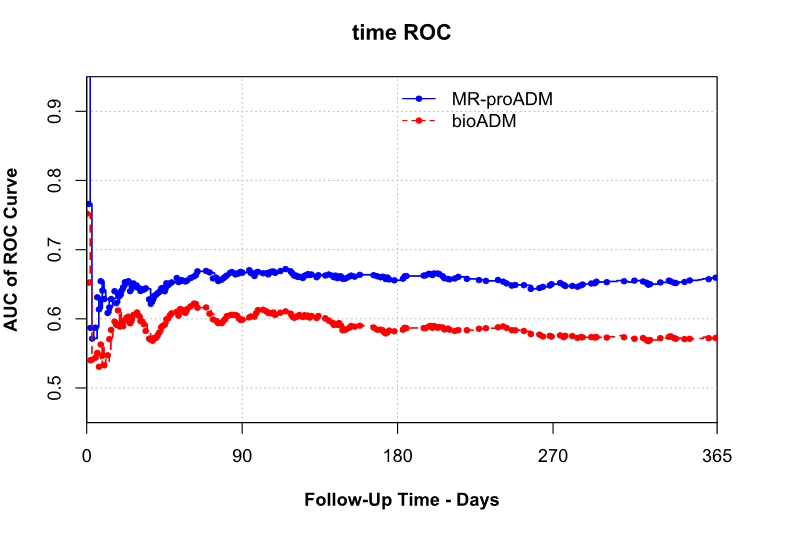


AHF: Acute heart failure. AUC of ROC curve: area under the time dependent receiver operating characteristic curve. bio-ADM: bioactive adrenomedullin. MR-proADM: midregional proadrenomedullin.

**Supplemental Figure 16.** All-cause mortality according to bio-ADM and MR-proADM concentrations at presentation and discharge after excluding patients receiving palliative care. A) Values at admission, B) Values at discharge. Bio-ADM levels were available for 1,876 patients at admission and 991 patients at discharge. MR-proADM levels were available for 758 patients at admission and 435 patients at discharge.


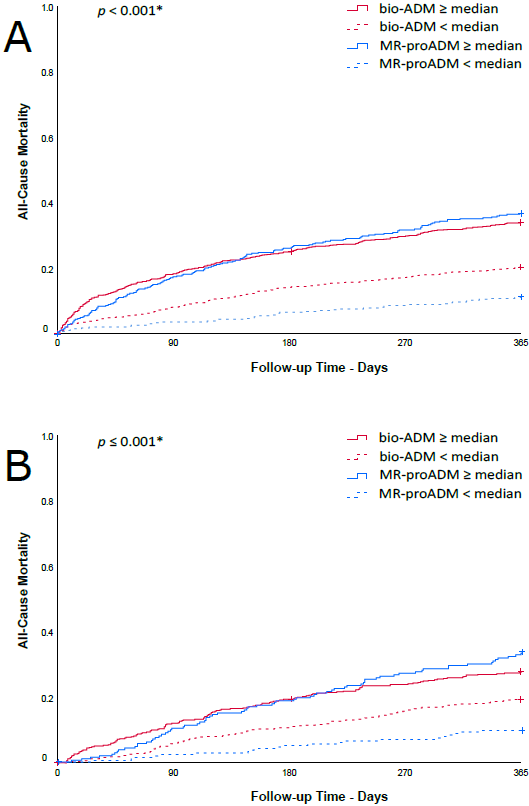


* for the comparison between bio-ADM groups; for the comparison of MR-proADM groups, the *p*-value was <0.001 in both A) and B)

bio-ADM: bioactive adrenomedullin. MR-proADM: midregional proadrenomedullin.
